# Two wrongs make a right: heat stress reversion of a male-sterile *Brassica napus* line

**DOI:** 10.1093/jxb/erac082

**Published:** 2022-02-28

**Authors:** Petra Schuhmann, Carina Engstler, Kai Klöpfer, Irene L Gügel, Amine Abbadi, Felix Dreyer, Gunhild Leckband, Bettina Bölter, Franz Hagn, Jürgen Soll, Chris Carrie

**Affiliations:** Department Biologie I–Botanik, Ludwig-Maximilians-Universität München, Großhadernerstr. 2–4, D-82152 Planegg-Martinsried, Germany; Department Biologie I–Botanik, Ludwig-Maximilians-Universität München, Großhadernerstr. 2–4, D-82152 Planegg-Martinsried, Germany; Bavarian NMR Center (BNMRZ) at the Department of Chemistry, Technical University of Munich, Lichtenbergstrasse 4, D-85748 Garching, Germany; Department Biologie I–Botanik, Ludwig-Maximilians-Universität München, Großhadernerstr. 2–4, D-82152 Planegg-Martinsried, Germany; Center of Advanced European Studies and Research (caesar), Ludwig-Erhard-Allee 2, D-53175 Bonn, Germany; NPZ Innovation GmbH, Hohenlieth-Hof, D-24363 Holtsee, Germany; NPZ Innovation GmbH, Hohenlieth-Hof, D-24363 Holtsee, Germany; Norddeutsche Pflanzenzucht Hans-Georg Lembke KG, Hohenlieth-Hof 1, D-24363 Holtsee, Germany; Department Biologie I–Botanik, Ludwig-Maximilians-Universität München, Großhadernerstr. 2–4, D-82152 Planegg-Martinsried, Germany; Bavarian NMR Center (BNMRZ) at the Department of Chemistry, Technical University of Munich, Lichtenbergstrasse 4, D-85748 Garching, Germany; Institute of Structural Biology, Helmholtz Zentrum München, Ingolstädter Landstraße 1, D-85764 Neuherberg, Germany; Department Biologie I–Botanik, Ludwig-Maximilians-Universität München, Großhadernerstr. 2–4, D-82152 Planegg-Martinsried, Germany; Munich Centre for Integrated Protein Science, CIPSM, Ludwig-Maximilians-Universität München, Feodor-Lynen-Str. 25, D-81377 Munich, Germany; Department Biologie I–Botanik, Ludwig-Maximilians-Universität München, Großhadernerstr. 2–4, D-82152 Planegg-Martinsried, Germany; Dipartimento di Biotecnologie, Università di Verona, Strada Le Grazie 15, 37134 Verona, Italy; School of Biological Sciences, University of Auckland, 3A Symonds Street, Auckland, 1142, New Zealand; University of Nottingham, UK

**Keywords:** *Brassica napus*, chloroplasts, fertility restorer, heat stress, male sterility, thermotolerance

## Abstract

Male-sterile lines play important roles in plant breeding to obtain hybrid vigour. The male sterility Lembke (MSL) system is a thermosensitive genic male sterility system of *Brassica napus* and is one of the main systems used in European rapeseed breeding. Interestingly, the MSL system shows high similarity to the 9012AB breeding system from China, including the ability to revert to fertile in high temperature conditions. Here we demonstrate that the MSL system is regulated by the same restorer of fertility gene *BnaC9-Tic40* as the 9012AB system, which is related to the translocon at the inner envelope membrane of chloroplasts 40 (TIC40) from Arabidopsis. The male sterility gene of the MSL system was also identified to encode a chloroplast-localized protein which we call BnChimera; this gene shows high sequence similarity to the sterility gene previously described for the 9012AB system. For the first time, a direct protein interaction between BnaC9-Tic40 and the BnChimera could be demonstrated. In addition, we identify the corresponding amino acids that mediate this interaction and suggest how BnaC9-Tic40 acts as the restorer of fertility. Using an RNA-seq approach, the effects of heat treatment on the male fertility restoration of the C545 MSL system line were investigated. These data demonstrate that many pollen developmental pathways are affected by higher temperatures. It is hypothesized that heat stress reverses the male sterility via a combination of slower production of cell wall precursors in plastids and a slower flower development, which ultimately results in fertile pollen. The potential breeding applications of these results are discussed regarding the use of the MSL system in producing thermotolerant fertile plants.

## Teaser

We have identified Tic40 as the restorer gene for the MSL system and hypothesize that heat treatment can also restore fertility via a cell cycle-induced developmental slow down.

## Introduction

In the coming century, predictions say that climate change will cause the earth’s temperature to increase by 3.7 °C (±1.1 °C) (Pachauri *et al.*, 2014). Since most processes which control plant growth and development are known to be heat sensitive, not surprisingly elevated temperatures are regularly attributed to cause yield reductions in economically important crops (Peng *et al.*, 2004; Zhao *et al.*, 2017; Tigchelaar *et al.*, 2018; Dusenge *et al.*, 2019; Jagadish, 2020). The most heat-sensitive stages of plant growth involve key processes of reproductive organ development, with pollen development considered as the most susceptible (Lohani *et al.*, 2020). Pollen function can be disrupted by heat stress in different ways, including reactive oxygen species (ROS) imbalance, differential hormone regulation (Ozga *et al.*, 2017), premature pollen development (Parish *et al.*, 2012), changes in carbohydrate and lipid metabolism (Jain *et al.*, 2010), tapetal cell abnormalities (Ku *et al.*, 2003), and disruption of meiotic cell division (Endo *et al.*, 2009). While heat stress causes male sterility, there are now a few male-sterile plant lines which, in fact, are male fertile after heat stress or when grown at higher than normal growth temperatures (Zhu *et al.*, 2010; Fernandez-Gomez *et al.*, 2020). Understanding how heat stress can reverse male sterility may offer important clues about ways of engineering plants able to survive and reproduce in the current warming climate.

In general, male sterility is not a positive trait for any organism, but it plays a significant role in plant breeding, especially in crop production, to achieve heterosis. Heterosis (or hybrid vigour) describes the outperformance of the hybrid generation in comparison with its two parental lines. Heterosis can result in grain yield increases ranging from 20% to >50% (Tester and Langridge, 2010). Therefore, since male sterility prevents self-pollination of outcrossing crops, it greatly simplifies crop breeding by ensuring cross-pollination. In rapeseed (*Brassica napus*), significant heterosis effects have been documented and studied (Sernyk and Stefansson, 1983; Grant and Beversdorf, 1985). Presently, several male sterility systems exist, which are used as the main pollination control systems in rapeseed hybrid breeding, namely cytoplasmic male sterility (CMS), cytoplasmic induced male sterility (CIMS), self-incompatibility (SI), chemical hybridization agent (CHA), and genetic male sterility (GMS). Two recessive GMS systems, the 9012AB (also known as 7365ABC) system and the natural male sterility Lembke (MSL) system, have been extensively used in hybrid breeding in China and Europe, respectively (Frauen *et al*., 2003, 2006; Deng *et al.*, 2016). Interestingly, not only have both lines been reported to show fertility reversion upon heat treatment, but they also share potentially similar loci regulating male sterility.

Originally, the male sterility of the 9012AB system was thought to be caused by the interaction between three independent loci (*BnMs3*, *BnMs4*, and *BnRf*). However, more recent work has demonstrated that *BnMs4* is most probably allelic to *BnRf* (Dong *et al.*, 2012). Therefore, the male sterility of 9012AB is now understood to be conferred by *BnMs3/Bnms3* and the multiallelic *BnRf* locus, of which the male-sterile allele is *BnRf*^*b*^ (Dong *et al.*, 2012; Dun *et al.*, 2014; Xia *et al.*, 2016). After intense mapping, *BnMs3* was identified as a homologue of Tic40 (translocon of the inner envelope membrane of chloroplasts 40), a protein required for protein import into chloroplasts (Chou *et al*., 2003, 2006; Dun *et al*., 2011, 2014; Li *et al.*, 2012; Lee and Hwang, 2019). More recently, the male-sterile allele (*BnRf*^*b*^) was demonstrated to be a newly formed chimeric gene, which is located to either the chloroplast or the nucleus (Deng *et al.*, 2016; Xia *et al.*, 2016). The *BnRf*^*b*^ chimeric gene encodes a protein consisting of several segments and shows, among others, a sequence similarity to the Arabidopsis mitochondrial Hsp70 (Deng *et al.*, 2016; Xia *et al.*, 2016). The introduction of *BnRf*^*b*^ into Arabidopsis plants results in complete male sterility (Z. Zhang *et al.*, 2020). A mechanism was proposed, whereby BnRf^b^ interacts with the E3 ligase BRUTUS (BnaBTS) on the outer envelope membrane of chloroplasts, which disrupts the normal ubiquitin–proteasome system causing toxic effects (Z. Zhang *et al.*, 2020). These toxic effects are then possibly compensated by an interaction between BnMs3 and Toc33 (translocon of the outer envelope membrane of chloroplasts 33) or by environmental heat shock (Z. Zhang *et al.*, 2020). To date, no direct interaction between the restorer BnMs3 protein and the male sterility-causing protein BnRf^b^ protein has been observed (Xia *et al.*, 2016).

This study utilized the natural rapeseed MSL system, which was discovered by the Norddeutsche Pfanzenzucht Hans-Georg Lembke (NPZ) breeding company and which is one of the main male-sterile lines used in hybrid production in Europe (Frauen *et al*., 2003, 2006). Previous work on this system demonstrated that it shares many similarities with the 9012AB system used in China. For example, both lines initiate pollen abortion during the microsporocyte stage, both lines display a premature or retarded degradation of the tapetum, interestingly even though the two lines contain different cytoplasm types they share the same temporary maintainer system, and both lines display a heat shock male fertility restoration (Luo *et al.*, 2018). Intriguingly, the MSL system is also regulated by two genes: the restorer gene was identified as *BnaC9-Tic40*, which is the same gene that was identified for *BnMs3* (Dun *et al*., 2011, 2014), while the male sterility gene has not yet been defined.

In this work, the male sterility gene of the MSL system was determined to be a gene with high sequence similarity to *BnRf*^*b*^ from the 9012AB system. The proposed male sterility gene, now called *BnChimera*, was cloned and found to encode a chloroplast-targeted protein, which, when transformed into Arabidopsis, resulted in male-sterile flowers. We also demonstrated, in contrast to the work on the 9012AB system, that BnChimera directly interacts with the BnaC9-Tic40 restorer. One major quest of this study was to determine how heat treatment results in the reversion of male fertility in the MSL system. Here, we used RNA sequencing (RNA-seq) to identify target genes contributing to, firstly, the male sterility in the MSL lines and, secondly, to the reversion to male-fertile flowers after heat shock. Combining the RNA-seq experiment with previous published results of the 9012AB system, we propose that during the heat treatment, pollen development is stalled or potentially slowed down during pollen meiosis, which provides time for the affected plastids to produce enough fatty acids for the lipids of the pollen cell wall. This describes a contrasting but similar mechanism to that proposed for how cold treatment slowed development and reverted other temperature-sensitive genic male sterility (TGMS) lines to being male fertile (C. Zhang *et al.*, 2020; J. Zhu *et al.*, 2020).

## Materials and methods

### Plant material and growth conditions

All seeds for the MSL lines, including C545, SORA 1, A-Line, and B-Line, were provided by the NPZ (Hohenlieth, Germany). The C545 line represents a male-sterile line, which is derived from crossing the male-sterile A-line and the male-fertile maintainer B-line. SORA 1 is a commercially available male-fertile line, which comes from the MSL system. *Brassica napus* plants were grown under greenhouse conditions [standard: 16 h light (200 μmol m^-2^ s^-1^, 23 °C), 8 h dark (18 °C)] until flowering. For the heat stress treatment, plants were placed in a Percival chamber for 3 d at 37 °C with a day/night rhythm of 20 h light and 4 h night. The humidity level was maintained at ~80–90%. These conditions were chosen as they provided the best laboratory conditions for the restoration of fertility. Neither day length nor humidity alone could restore fertility. Humidity was kept high to prevent plants from drying out. Arabidopsis plants were grown under controlled long-day conditions [16 h light (100 μmol m^-2^ s^-1^, 22 °C), 8 h dark (18 °C), and 50% relative humidity] until flowering and then moved to a greenhouse. *Nicotiana benthamiana* plants were grown under standard greenhouse conditions.

### Transient expression of fluorescent proteins in *Nicotiana benthamiana*

The full coding sequence of the *BnChimera* gene or only the sequence encoding the very N-terminal 545 amino acids were combined as N-terminal green fluorescent protein (GFP) fusions via Goldengate cloning (Binder *et al.*, 2014; Chiasson *et al.*, 2019). For the transient expression of the respective fluorescent proteins, leaves of 4- to 6-week-old *N. benthamiana* plants were infiltrated with *Agrobacterium*, and isolation of protoplasts was performed as described (Schweiger and Schwenkert, 2014). Fluorescence signals were detected by confocal laser scanning microscopy (Leica TCS SP5) as previously described (Schweiger and Schwenkert, 2014).

### Stable transformation of Arabidopsis

For the assessment of the functionality of BnChimera in Arabidopsis, the full coding sequence of *BnChimera* and various truncated versions were assembled under the control of the putative native promoter of *BnChimera* via Goldengate cloning (Binder *et al.*, 2014; Chiasson *et al.*, 2019). The stable transformation was performed using *Agrobacterium* and the floral dip method (Clough and Bent, 1998). Positively transformed plants were selected by spraying with Basta^©^ and confirmed by genotyping using the primers listed in Supplementary Table S1.

### Pollen staining

*Brassica napus* pollen were stained for viability using acetocarmine solution (1% acetocarmine in 45% acetic acid which was first refluxed for 24 h and then filtered) (Heslop-Harrison, 1992). Pollen were examined under a light microscope (Leica DM1000) after staining.

### Yeast two-hybrid assays

Yeast two-hybrid assays were performed using the Matchmaker Gold Yeast two-hybrid system (Clontech). All genes were cloned without predicted chloroplast-targeting peptides and predicted transmembrane domains. Bait plasmids (pGBK) of BnChimera and its truncated versions were cloned as fusions to the Gal4 DNA-binding domain via Goldengate cloning and transformed into the Y2HGold yeast strain (Chiasson *et al.*, 2019). Prey plasmids (pGAD) of the various *B. napus* Tic40-like and BnaC9-Tic40 point mutations were cloned as fusions to the Gal4 activation domain via Goldengate cloning and transformed into the yeast strain Y187 (Chiasson *et al.*, 2019). Each bait and prey combination was mated and plated first on selection medium lacking the amino acids leucine and tryptophan (-Leu,-Trp). To test for positive interactions, positively mated combinations were inoculated overnight cultures in liquid -Leu,-Trp medium. Overnight cultures were then diluted and spotted onto -Leu,-Trp solid medium or medium lacking leucine, tryptophan, adenine, and histidine (-Leu,-Trp,-Ade,-His). Growth on -Leu,-Trp,-Ade,-His medium indicated a positive interaction. For positive and negative controls, the murine p53 and Lamin (Lam) were mated with the SV40 large T-antigen, respectively (Li and Fields, 1993). For the analysis of protein expression in yeast, immunoblotting was performed on selected colonies. Colonies of mated interaction partners were inoculated in -Leu,-Trp medium and grown overnight at 30 °C. Cells were pelleted by centrifugation (700 *g*, 5 min), washed in 1 mM EDTA, and resuspended in 2 M NaOH. The same volume of 50% trichloroacetic acid (TCA) was added to NaOH. After centrifugation (14 000 *g*, 20 min, 4 °C), ice-cold acetone was used to wash the pellet. The centrifugation step was repeated, and 5% SDS and the same volume of SDS loading buffer were added to the pellet. The cells were vortexed and Tris base added if the sample buffer turned yellow. The yeast extract was incubated (37 °C, 15 min, shaking), centrifuged (14 000 *g*, 15 min), and the supernatant used directly for SDS–PAGE or stored at –20 °C until use.

### Isolation of genomic DNA of *B. napus*

Genomic DNA isolation of *B. napus* leaves was done with the innuPREP Plant DNA kit (Analytikjena, Jena, Germany) according to the manufacturer’s instructions.

### RNA isolation from *B. napus*

RNA was isolated from unopened flower buds of 2 mm from the indicated genotype and treatments using the RNeasy Plant Mini Kit (Qiagen) according to the manufacturer’s instructions. Plants were grown in three separate batches with several buds taken from different plants from each batch representing the three replicates used in the RNA-seq for each genotype and temperature treatment. RNA was DNase treated using the Turbo DNase I kit (Ambion) according to the manual. RNA integrity was assayed via agarose gel electrophoresis and quantified using a Nanodrop.

### RNA-seq

Preparation of RNA-seq libraries (three replicates per genotype and temperature treatment) and sequencing using 150 bp paired-end mode was performed by Novogene Biotech (Beijing) using standard Illumina protocols. Quality control was performed using the FastQC software. Transcript abundance as transcripts per million and estimated counts were quantified on the gene level against the previously published *B. napus* reference genome using Salmon (Chalhoub *et al.*, 2014; Patro *et al.*, 2017). Differentially expressed genes (DEGs) were calculated using the 3DRANseq pipeline and were selected based on a log2-fold change ≥1 or ≤ −1 with an adjusted *P*-value of ≤0.05 (Guo *et al.*, 2020). Gene Ontology (GO) term annotation of the *B. napus* genome was performed using Blast2GO (Conesa and Gotz, 2008). GO term enrichment analysis was performed using ClusterProfiler and enrichplot with a Holm–Bonferroni test correction (Yu *et al.*, 2012).

### Light microscopy

For microscopic analysis, we used 2 mm long unopened flower buds from the indicated genotypes and environmental treatments. Buds were fixed immediately after harvesting with 2.5% (w/v) glutaraldehyde (4 °C, at least 24 h) in 75 mM cacodylate buffer (2 mM MgCl_2_, pH 7.0), rinsed several times with fixative buffer, and subsequently post-fixed with 1% (w/v) osmium tetroxide for at least 2.5 h in fixative buffer at 20 °C. After five washing steps in distilled water, samples were stained with 1% (w/v) uranyl acetate in 20% acetone, dehydrated with a graded acetone series, and embedded in Spurr’s low viscosity epoxy resin (Spurr, 1969). For light microscopy, semi-thin sections (1–2 µm) were cut with a glass knife (Pyramitome 11800, LKB).

### qRT–PCR

The synthesis of cDNA from 500 ng of purified RNA (three replicates per genotype and temperature treatment) was performed with the iScript cDNA synthesis kit (BIORAD) as instructed by the manufacturer’s manual. For quantitative reverse transcription–PCR (qRT–PCR), a 20 μl reaction contained 10 μl of LightCycler FastStart Essential DNA Green Master mix (Roche), 2 μl of diluted cDNA (10-fold dilution in H_2_O), and 1 μM oligonucleotides (Supplementary Table S1). The reactions were performed using the LightCycler96 (Roche) with the following program: one cycle at 95 °C for 60 s, 45 cycles of a three-step amplification (95 °C for 10 s, 60 °C for 10 s, 72 °C for 10 s), and finally a melting curve was performed. The 2^ΔΔ–ct^ method was used to calculate fold changes (Pfaffl, 2001). Both BnActin (BnaA02g00190D) and Tic40 (BnaA02g03180D) were used as reference genes for normalization using the method outlined in Hellemans *et al.* (2007), as they were found independently from the RNA-seq data to not change significantly in the conditions used.

### Protein production

Recombinantly produced ^15^N-labelled Bna-Tic40 variants A10 and C9, as well as ^13^C,^15^N-labelled A10 were purchased from CRELUX (Martinsried, Germany) and used for all spectroscopic experiments at the indicated concentrations and buffer conditions.

### Circular dichroism (CD) spectroscopy

Far-UV CD spectra were recorded with 20 µM protein samples in 10 mM NaPi pH 6.0, 20 mM NaCl, 0.5 mM EDTA, and 2 mM β-mercaptoethanol with a Jasco J-715 spectropolarimeter (Jasco, Pfungstadt, Germany) at 20 °C. Raw ellipticity data were converted to mean residue ellipticity (Kelly *et al.*, 2005). For the titration with trifluoroethanol (TFE), a series of separate samples were prepared containing 0, 5, 10, or 20% (v/v) TFE.

### NMR spectroscopy

NMR experiments were conducted with a 600 MHz spectrometer (Bruker Biospin) equipped with a cryogenic probe. 2D-[^15^N,^1^H]-HSQC NMR spectra of 400 µM ^15^N-labelled BnaA10- and BnaC9-Tic40 protein samples in 20 mM NaPi pH 6.0, 50 mM NaCl, 0.5 mM EDTA, 1 mM TCEP, 7% D_2_O were recorded with 32 scans per increment and 256 complex points in the indirect 15N dimension at 303 K and in the presence of 10% (v/v) *d*_3_-trifluoroethanol. For backbone resonance assignment of A10 (residues 305–457), a set of 3D-triple resonance experiments (Sattler *et al.*, 1999), consisting of HNCO, HN(CA)CO, HNCA, HN(CO)CA, HNCACB, and CBCA(CO)NH, were recorded with a 1.1 mM ^13^C,^15^N-labelled Tic40 A10 sample at 308 K in 20 mM NaPi pH 6.0, 50 mM NaCl, 0.5 mM EDTA, 1 mM TCEP, 0.02% sodium azide, 10% (v/v) *d*_3_-trifluoroethanol. Data analysis and resonance assignment was done with NMRFAM-SPARKY (Lee *et al.*, 2015).

### Structure prediction

A structural model of *Arabidopsis thaliana* Tic40 (residues 298–447) was obtained from the AlphaFold Databank (https://alphafold.ebi.ac.uk) (Jumper *et al.*, 2021; Tunyasuvunakool *et al.*, 2021) and validated with experimental NMR data as well as a previously determined NMR structure of the C-terminal NP domain of Tic40 (Kao *et al.*, 2012).

## Results

### Heat stress restoration of fertility of the *Brassica napus* male-sterile C545 line

**Fig. 1.**
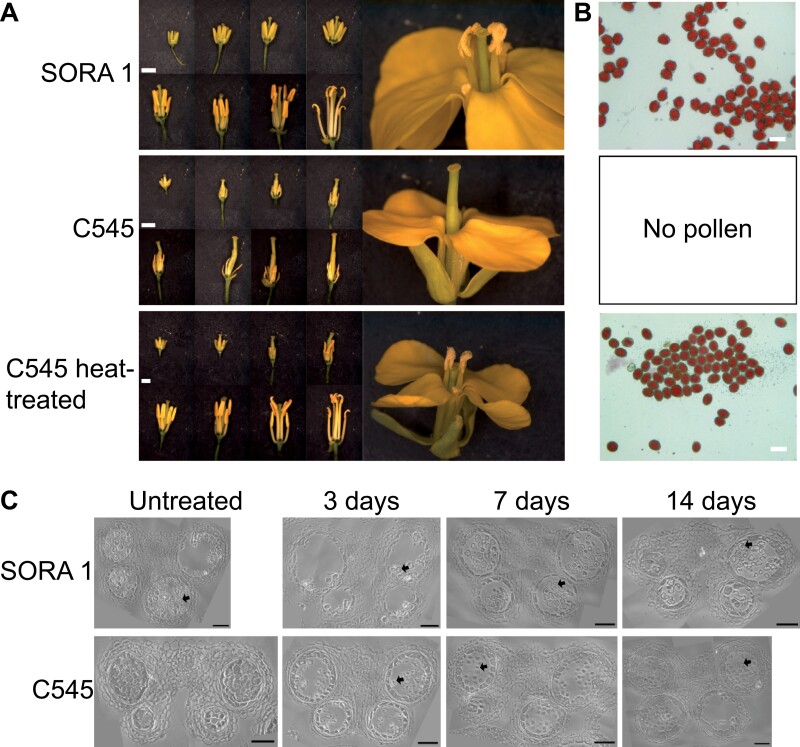
Heat treatment restores fertility of the C545 male-sterile Brassica napus line. (A) Anther and flower development of the restorer line SORA 1, the male-sterile line C545, and C545 after 3 d of heat treatment at 37 °C. Shown are dissected flowers starting from 0.3 cm to 1 cm in length. Scale bar=2 mm. (B) Acetocarmine staining of the indicated lines displaying viable pollen if stained red. C545 untreated plants do not produce pollen, so are not shown. Scale bar=50 µm. (C) Light microscopy images of semi-thin sections (1–2 µm) from representative anthers for 2 mm long buds from SORA 1 and C545 lines. Buds are at stages 9–10 of development. The number of days indicates the duration of heat treatment at 37 °C. Arrows indicate released mature microspores. Scale bar=50 µm.

Under standard growth conditions, the C545 line displays normal vegetative growth, but typically presents a male-sterile phenotype (Fig. 1A). The male-sterile phenotype was characterized as flowers with shorter filaments and severely degraded anthers, completely devoid of pollen (Fig. 1A). However, upon short heat treatment at 37 °C, male fertility could be restored (Fig. 1A). The flowers now exhibit normal anthers, which contained viable pollen, visualized by a red staining with acetocarmine (Fig. 1B). Previous work on similar *B. napus* male-sterile lines had demonstrated that male sterility was caused via exaggerated vacuolation and abnormal expansion of the tapetal cells, which crushed the developing tetrads (Wan *et al.*, 2010; Zhu *et al.*, 2010; Dun *et al.*, 2011). This led to an inability to distinguish middle layer cells from the others at approximately stage 9 of anther development. In stage 9 of anther development, microspores generate the exine wall and become vacuolated, which is followed by stage 10 with the initiation of tapetum degeneration (Sanders *et al.*, 1999). To observe what occurs inside the anthers, 2 mm long buds were selected and analysed under light microscopy. Flower buds with a length of 2 mm should represent anthers at approximately developmental stages 9–10. Accordingly, in the SORA 1 control line, free microspores are readily visible. However, in the untreated C545 line, microspores cannot be distinguished from the surrounding tapetal cells (Fig. 1C; Supplementary Fig. S1). This is consistent with previous results demonstrating that the tetrads were typically surrounded with thick callose, which is degraded in male-fertile plants (Wan *et al.*, 2010; Zhu *et al.*, 2010; Dun *et al.*, 2011). Anthers were also analysed after heat treatment at 37 °C for 3, 7, and 14 d, respectively (Fig. 1C; Supplementary Fig. S1). Even though in the C545 line the tapetal cell layer may still be enlarged compared with the fertile SORA 1 line, fully developed microspores can be observed (Fig. 1C; Supplementary Fig. S1). These results demonstrate that male fertility can be (partially) restored in the C545 line via a short 3 d heat treatment at 37 °C and that the physiological causes of this male sterility are consistent with previous reports of similar breeding systems (Wan *et al.*, 2010; Zhu *et al.*, 2010; Dun *et al.*, 2011).

### The restorer gene of the MSL system is *BnaC9-Tic40*

To determine the restorer gene of the MSL system, crosses between a male-sterile MSL A-line (*rr*) and fertile restorer line (*RR*) and subsequent selfing of the fertile F_1_ plants resulted in a large F_2_ progeny (*n*=2000) that were used for phenotyping of fertility/sterility and fine mapping of the Restorer (*Rf*) gene. Furthermore, a BAC (bacterial artificial chromosome) library generated from the above-mentioned MSL A-Line (*rr*) was screened to this end with simple sequence repeat- (SSR) flanking *Rf* markers (HMR0875, HMR1750, and HMR1882) to identify BAC clones harbouring the *rf* gene. At each step, screening of the BAC library was accompanied by phenotypic evaluation of the F_2_ progeny to identify recombinant genotypes and validate the markers mentioned above.

Two BAC clones (BAClone27 and BAClone3) fished with the marker HMR0875 were subjected to Roche 454 Next Generation sequencing (Rx Biosciences, Ltd). After assembly, 236 contigs with lengths varying between 220 bp and 11 328 bp were recovered from the BAClone3 and subjected to physical mapping with the *Rf* markers HMR0875, HMR1750, and HMR1882 (Supplementary Fig. S2) to identify linkage and recombination events (Supplementary Table S2).

Contig_8 exhibited a tight linkage with the marker HMR1750 (no recombinants were found with this marker) and was then subjected to an ORF search to identify genes underlying the linked region, and to BLAST searches against The Arabidopsis Information Resource (TAIR; Huala *et al.*, 2001). The position of the marker HMR1750 was identified to be on the ORF of a Tic40-like protein (At5g16620, BnaC9-Tic40) representing a small 2 bp deletion leading to a frameshift and truncated protein (Supplementary Fig. S2). Single nucleotide polymorphism (SNP) markers differentiating between the mutant (short Tic40, *rr*) and restorer gene (native Tic40, *RR*) were designed and validated on a large set of *rr*, *Rr*, and *RR B. napus* genotypes. The 2 bp deletion in *BnaC9-Tic40* was confirmed via PCR and Sanger sequencing using the RCP170 primer pair (Supplementary Fig. S2).

### The male sterility of C545 is caused by a chloroplast-targeted chimeric protein

**Fig. 2.**
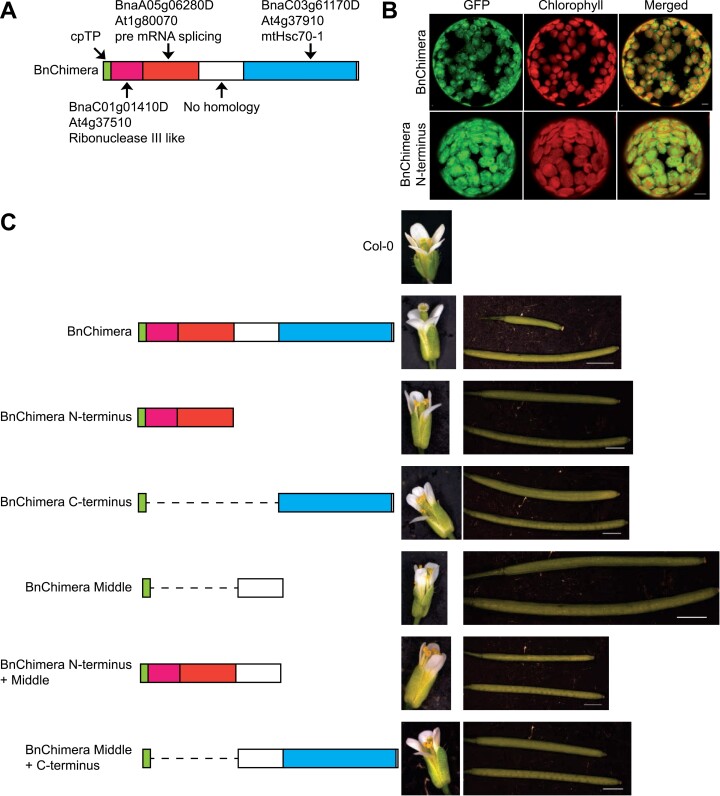
The *MS* gene is a chimeric gene targeted to the chloroplasts, causing male sterility in Arabidopsis. (A) Schematic diagram of the protein domains encoded by the *MS* gene, also called *BnChimera*. The first 42 amino acids contain a predicted chloroplast-targeting peptide. Amino acids 41–214 display a homology to At4g37510, encoding an RNase III-like protein (coloured orange). Amino acids 214–518 display homology to At1g80070, encoding SUS2, which is required for pre-mRNA splicing (coloured red). Amino acids 519–761 display no homology to any known protein (coloured white). Amino acids 762–1375 display homology to At4g37910, which encodes a mitochondrial Hsp70 (mtHsc70-1, coloured blue). (B) Protoplasts of transiently transformed tobacco leaves expressing either the full coding sequence or the N-terminus of BnChimera tagged to the N-terminus of GFP, monitored by laser scanning microscopy. Chlorophyll autofluorescence (red channel) indicates the location of chloroplasts. Scale bar=5 µm. (C) On the left are schematic diagrams of the various constructs of BnChimera under the control of the putative *BnChimera* promoter transformed into Arabidopsis. On the right are representative images of the phenotypes observed for the flowers and siliques after transformation with either construct. A typical wild type (Col-0) is shown as a control. Scale bars=2 mm except for the BnChimera siliques photo, where it represents 3 mm.

Due to the phenotypic and genetic similarities in the observed male sterility of C545 and knowing that BnaC9-Tic40 could act as a restorer gene for male fertility, we reasoned that the *MS* gene is most probably also the same or similar to that of recently characterized systems (Li *et al.*, 2012; Deng *et al.*, 2016; Xia *et al.*, 2016). The putative *MS* gene of the MSL system was also mapped to a region on chromosome A07, which is very close to the regions recently described by Deng and Xia and their co-authors (Deng *et al.*, 2016; Xia *et al.*, 2016). In both cases, a similar chimeric gene was reported: amino acids 41–214 display a homology to the Arabidopsis At4g37510, which encodes an RNase III-like protein; amino acids 214–518 display a homology to At1g80070, which encodes ABNORMAL SUSPENSOR 2 (SUS2) or PRE-RNA PROCESSING 8 (PRP8) and which is a conserved member of the spliceosome (Grainger and Beggs, 2005); amino acids 762–1375 display a homology to At4g37910, which encodes a mitochondrial Hsp70 (mtHsc70-1); and amino acids 519–761 display no homology to any known protein (Deng *et al.*, 2016; Xia *et al.*, 2016) (Fig. 2A). The only major difference between the two previous published works is that Xia *et al.* (2016) localized the protein to chloroplasts whereas Deng *et al.* (2016) detected it in the nucleus. Therefore, we first determined if this same chimeric gene could also be found in the MSL system. For this, the three primer sets HY1, HY2, and HY3, respectively, described by Xia *et al.* (2016) were used for PCR amplifications on DNA isolated from the following lines from the MSL system: the male-sterile A-line and C545 line, the male-fertile maintainer B-line, and SORA 1, which is a male-fertile restorer line. Only for the A-line and C545 line did we obtain positive PCR fragments for all three primer pairs (Supplementary Fig. S3). These fragments were confirmed via sequencing and contained fragments identical to those identified previously (Deng *et al.*, 2016; Xia *et al.*, 2016). This indicates that the *MS* gene in the MSL system is most probably the same chimeric gene as described before (Deng *et al.*, 2016; Xia *et al.*, 2016).

Since the subcellular localization of this chimeric protein (from now on for simplicity referred to as BnChimera) is currently in dispute, we first investigated if it is a chloroplast protein like the restorer BnaC9-Tic40. To determine the subcellular location of BnChimera, two different GFP constructs were generated. The first construct contained the full coding sequence of BnChimera fused to the N-terminus of GFP. The second contained the first 545 N-terminal amino acids (BnChimera N-terminus) fused to the N-terminus of GFP. A transient expression of both constructs in tobacco epidermal cells showed a clear chloroplast localization, as evidenced by the overlap with chlorophyll autofluorescence (Fig. 2B). This clearly demonstrates that both the restorer gene *BnaC9-Tic40* and *BnChimera* code for proteins targeted to the chloroplast. Here, it must be mentioned that the GFP signals of both BnChimeria constructs were consistent with either a stromal or a thylakoid localization within chloroplasts and not in the envelope membranes. This observation is important when interpreting the possible mode of action of BnChimera.

If *BnChimera* is in fact the *MS* gene, its protein should be able to confer male sterility on Arabidopsis plants. A plasmid was constructed that contained the *BnChimera* coding sequence under the control of its putative native promoter and transformed into Arabidopsis by floral dip. After selection of primary transformants with Basta®, positively transformed plants were allowed to grow normally. All the plants transformed with the *BnChimera* construct were male sterile and like the *Brassica* C545 flowers, as mature anthers failed to develop (Fig. 2C). Since Arabidopsis is self-pollinating, this also led to the development of extremely short empty siliques (Fig. 2C). This demonstrates that BnChimera can cause male sterility, indicating that it is the *MS* gene from the MSL system. We were then interested in which parts of the protein cause the male-sterile phenotype. To analyse this, five further constructs of the BnChimera were made: the N-terminus, C-terminus, middle, N-terminus+middle, and middle+C-terminus. None of these constructs resulted in male sterility in Arabidopsis (Fig. 2C), demonstrating that only the full-length protein of BnChimera is responsible for causing the male-sterile phenotype. A similar result was also observed recently by others (Z. Zhang *et al.*, 2020).

### BnChimera can directly interact with the restorer BnaC9-Tic40

**Fig. 3.**
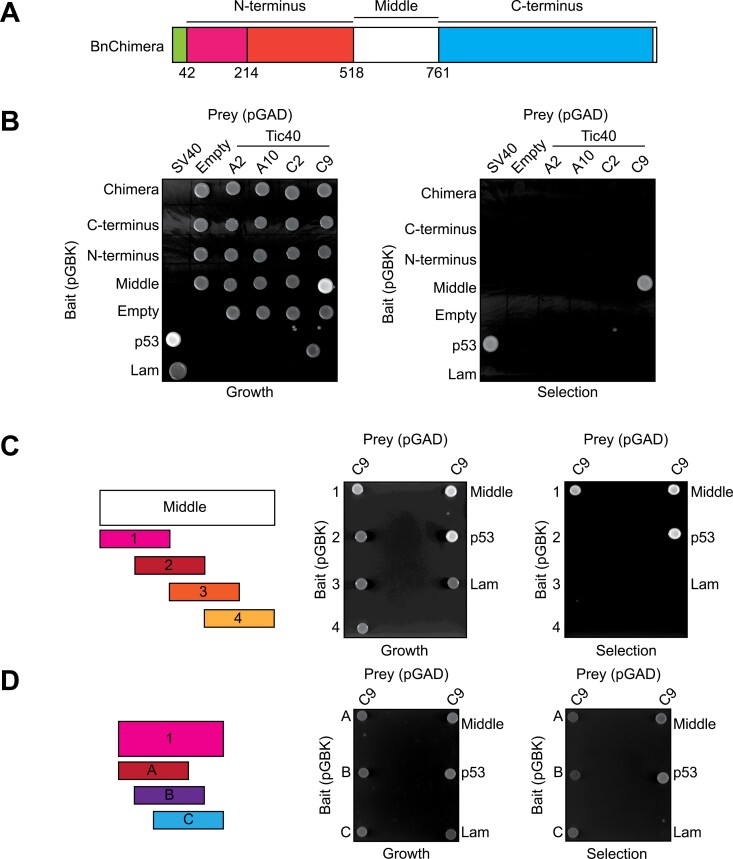
The middle non-homologous section of BnChimera directly interacts with BnaC9-Tic40. (A) Schematic diagram of the domains of BnChimera used in yeast two-hybrid assays. (B) Four versions of BnChimera (full sequence, N-terminus, middle, and C-terminus) were tested for interactions with the four Tic40 proteins (BnaA2, BnaA10, BnaC2, and BnaC9) from *Brassica napus*. Bait and prey plasmids were individually transformed into the correct yeast strains and combinations were mated. Mated yeast were then plated on either growth medium (SD medium -Leu,-Trp) to assay correct mating or on selection plates (SD medium -Leu,-Trp,-Ade,-His) to assay for positive interactions. Positive interactions appear as white colonies on the selection plates. For positive and negative controls, murine p53 and Lamin (Lam) were each mated with the SV40 large T-antigen. (C) The middle section, which positively interacted with BnaC9-Tic40, was divided into four overlapping fragments (1, 2, 3, and 4) and re-assayed for the interaction with BnaC9-Tic40. Yeast two-hybrid assays were performed as in (B). (D) Fragment 1 from the middle section of the BnChimera was again divided into three overlapping fragments (A, B, and C) and tested for interaction with BnaC9-Tic40 in the same manner as in (B).

Due to the observations that both the *BnChimera* and the restorer gene *BnaC9-Tic40* encode chloroplast-targeted proteins, we explored if their proteins can functionally interact. For this, we performed yeast two-hybrid assays, with the bait plasmids containing constructs from BnChimera and either of its three individual sections (N-terminus, middle, or C-terminus) and prey plasmids containing either of the four Tic40-like proteins identified in the *B. napus* genome (Fig. 3). To ensure proper expression, all chloroplast-targeting signals and transmembrane domains were removed in the bait and prey plasmid constructs. Apart from the positive control, the only combination of bait and prey plasmids which resulted in a positive interaction was with the BnChimera middle section and BnaC9-Tic40 (Fig. 3B). While the full-length *BnChimera* bait construct should also code for the middle section, no interaction was observed. Further analysis revealed that the full-length BnChimera is not expressed in yeast, possibly due to its large size (Supplementary Fig. S4). So, the only Tic40 protein which positively interacts in yeast with BnChimera is BnaC9-Tic40, counter-intuitively interacting to the non-homologous middle section. This is in contrast with previous work where no interaction between BnaC9-Tic40 and BnChimera was observed (Xia *et al.*, 2016; Z. Zhang *et al.*, 2020).

To further determine the amino acids enabling the interaction of BnaC9-Tic40 with the BnChimera middle section, the middle section was divided into four overlapping parts (Fig. 3C). The yeast two-hybrid assay was then repeated solely against BnaC9-Tic40 (Fig. 3C). In this instance, section 1 was the only section interacting with BnaC9-Tic40 (Fig. 3C). In addition, when this section 1 was further divided into three more overlapping sections, part A showed the strongest interaction (Fig. 3D), indicating that these approximate 50 amino acids are the most important for the interaction between BnChimera and BnaC9-Tic40.

**Fig. 4.**
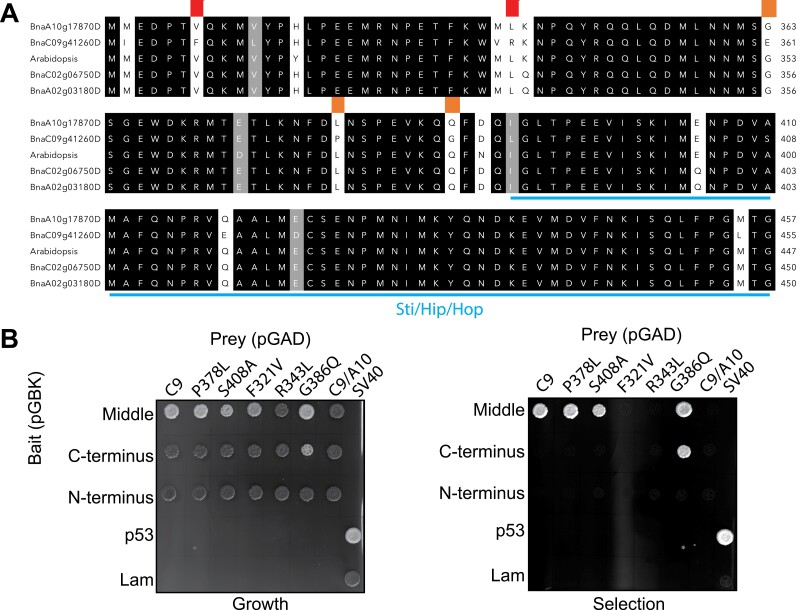
Amino acids F321 and R343 of BnaC9-Tic40 are essential for the interaction with BnChimera. (A) Multiple sequence alignment of the C-termini of the four *B. napus* Tic40 proteins and of Arabidopsis Tic40. Identical amino acids are highlighted in black and similar amino acids in grey. The Sti/Hip/Hop domain is highlighted in blue. Red highlighted amino acids represent essential amino acids for the interaction between BnaC9-Tic40 and the middle fragment of BnChimera. Orange highlighted amino acids represent amino acids which are different only in the BnaC9-Tic40 but which are not essential for the interaction with BnChimera. (B) Yeast two-hybrid assays to determine essential amino acids required for the interaction of BnaC9-Tic40 with the BnChimera middle fragment. Yeast two-hybrid assays were performed as in Fig. 3.

Since the *B. napus* genome encodes for four different Tic40-like proteins and only BnaC9-Tic40 interacts in yeast with BnChimera, and since it is also responsible for the restoration of fertility, we next sought to identify the difference between BnaC9-Tic40 and the other three Tic40-like proteins. A multiple sequence alignment demonstrates that the C-termini of the four Tic40-like proteins are highly similar (Fig. 4A). Interestingly, only five previously described amino acids are unique to BnaC9-Tic40 and are present in all plant species but not in the other three *B. napus* Tic40-like proteins (Fig. 4A) (Dun *et al.*, 2014). To test the functionality of these five amino acids in BnaC9-Tic40, a site-directed mutagenesis was performed followed by a repetition of the yeast two-hybrid assays (Fig. 4B). In all cases, the amino acids were mutated to a corresponding amino acid present in BnaA10-Tic40, which is the closest to BnaC9-Tic40. The interaction between BnaC9-Tic40 and the BnChimera middle section was abolished only if amino acids 321 (F321V) and 343 (R343L) were mutated (Fig. 4B). Interestingly, Z. Zhang *et al.* (2020) also demonstrated that a similar mutation of amino acid 321 abolished the restorer activity of the BnaC9-Tic40 protein.

### CD and NMR studies of BnaC9 and BnaC10-Tic40 proteins

**Fig. 5.**
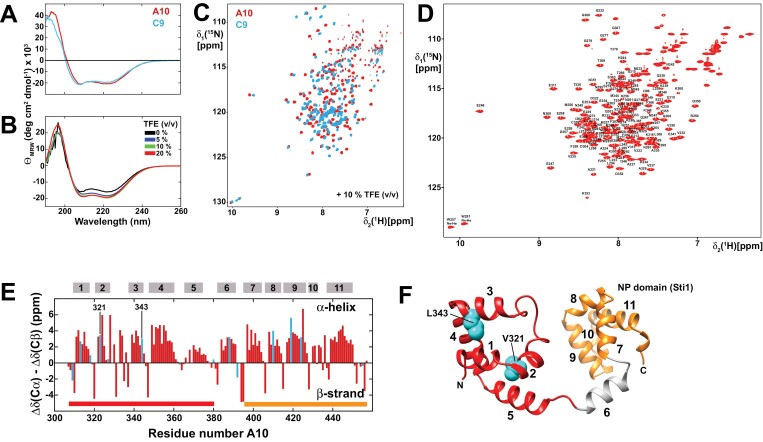
Biophysical analysis of two *B. napus* Tic40 variants and secondary structure determination of BnaA10-Tic40. (A) Far-UV CD spectra of BnaA10-Tic40 (red) and BnaC9-Tic40 (blue) indicate an α-helical secondary structure in both cases. (B) Addition of trifluoroethanol (TFE) to A10 leads to an increase in the secondary structure content as monitored by far-UV CD. No further difference could be detected between 10% and 20% TFE. (C) 2D-[^15^N,^1^H]-HSQC NMR spectra of A10 and C9 suggest structural differences between the two Tic40 variants. (D) 2D-[^15^N,^1^H]-HSQC NMR spectrum of 1.1 mM ^13^C,^15^N-labelled BnaA10 + 10% (v/v) TFE at 308 K with the assigned backbone amide resonances labelled. (E) ^13^C Secondary chemical shift information of BnaA10 indicates 11 α-helical secondary structure elements, marked by the grey boxes above. Positions that show sequence variations between A10 and C9 are labelled in blue. Residues that abolish binding to the BnChimera in A10 are labelled (numbering according to the C9 sequence). (F) Structural model of the Tic40 fragment (N-terminal domain in red, C-terminal NP domain in orange) derived with the program AlphaFold (Jumper *et al.*, 2021). The model for the C-terminal NP domain is in perfect agreement with the experimental NMR structure (Kao *et al.*, 2012) (2LNM). Between the two domains, an additional α-helical segment (helix 6) was detected by NMR and is also visible in the structural model.

The structures of the two Bna-Tic40 variants C9 and A10 were first analysed by far-UV CD spectroscopy (Fig. 5A). Both variants showed a typical spectrum indicative of an α-helical secondary structure. Since the initial NMR spectral quality of the C9 variant was not sufficient for a more detailed NMR analysis (Supplementary Fig. S5), we chose to use the A10 variant for further experiments. In order to improve the stability of the A10 variant, we added the α-helix-stabilizing solvent TFE and monitored the gain in secondary structure by CD spectroscopy. As shown in Fig. 5B, the addition of up to 10% TFE led to a gain in the α-helical secondary structure content, while higher TFE concentrations did not have any additional effect. Thus, we added 10% TFE to ^15^N-labelled A10 and C9 samples (Fig. 5C) and compared the NMR spectral signatures using 2D-[^15^N,^1^H]-HSQC experiments. The presence of TFE markedly improved the spectral qualities of both Tic40 variants. However, A10 still displays a much better spectral quality than the C9 variant. In these NMR spectra, the positions and the intensities of the signals are highly sensitive to changes in structure and dynamics. The analysis of the spectra shows a large number of changes in the signal positions as well as intensities originating from only a relatively low number of sequence variations between these two variants, suggesting that the structure and dynamics are altered.

In order to probe the structural state of the A10 variant that appeared to be most promising for a more detailed NMR analysis, we recorded a set of three-dimensional NMR experiments to obtain sequence-specific resonance assignments (Fig. 5D). The obtained ^13^C-NMR chemical shift information was further used to determine the location of the secondary structure elements in the protein (Fig. 5E). Only α-helical secondary structure could be determined with this methodology, in line with the CD data. Furthermore, the variant residue positions that lead to abolished binding to BnChimera (F321V, R343L) in A10 are both located in α-helical secondary structure elements.

Next, we utilized the recently developed software AlphaFold (Jumper *et al.*, 2021) to derive a structural model of the investigated Tic40 fragment (residues 305–457). The deposited model of Tic40 from *A. thaliana*, showing high sequence identity with Bna-Tic40, was in very good agreement with the location of the α-helical secondary structural elements obtained with NMR (Fig. 5F). Furthermore, the available NMR solution structure of the C-terminal NP domain of Tic40 (Kao *et al.*, 2012) from *A. thaliana* overlaid very well with the predicted structural model as well as the herein experimentally determined α-helical secondary structure elements. The elongated shape of the model and the lack of larger hydrophobic clusters, which would be expected for a compactly folded protein, are consistent with the relatively low NMR spectral quality. The two mutations that exhibited the most pronounced effect on the ability to bind to BnChimera are located in the N-terminal segment with position 321 oriented toward the interior, whereas position 343 appears to be partially surface exposed. Thus, mutation at these positions most probably has an impact on the folding state of the N-terminal segment. This is supported by the observed reduced NMR spectral quality for the C9 variant indicating that this protein exhibits a higher degree of flexibility and a weakening of the folding state.

These observations allow for the conclusion that the binding between BnaC9-Tic40 and the BnChimera to restore fertility depends either on interactions mediated by the specific amino acids F321 and R343 or, more globally, by the less compact folding state of C9 that might facilitate the exposure of hydrophobic surfaces.

### Transcriptomic analysis of C545 before and after heat treatment

**Fig. 6.**
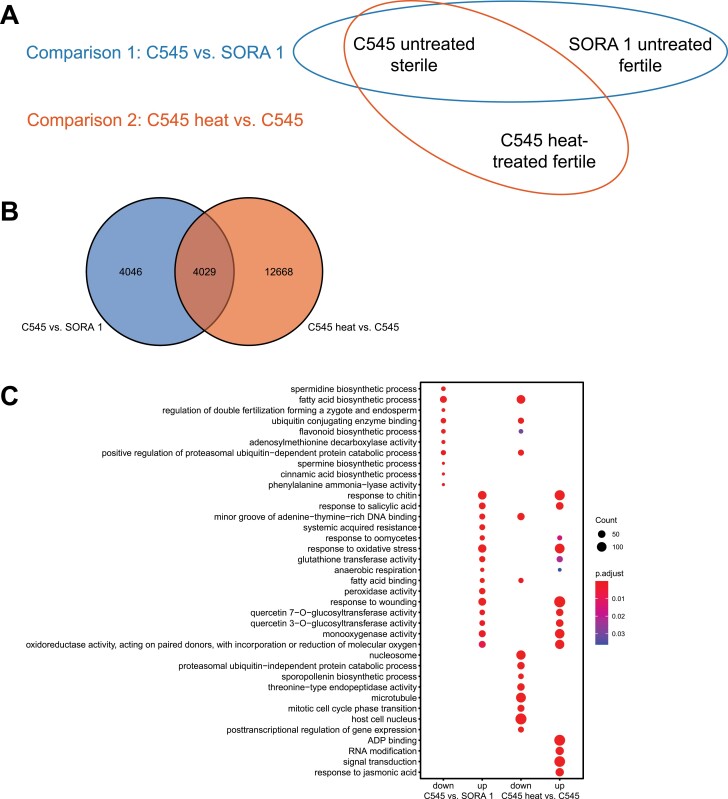
Experimental setup for the RNA-seq, analysis of differentially expressed genes (DEGs), and GO term enrichments in the two comparisons. (A) Overview of the strategy for RNA-seq and analysis of DEGs. RNA was isolated from 2 mm long buds, which should represent pollen development stages 9–10. This is the approximate stage where the sterile C545 line and the fertile SORA 1 line diverge. Two comparisons were performed. Comparison 1 was between the male-sterile C545 line and the fertile SORA 1 line to determine the causes of male sterility in C545. Comparison 2 investigated differences in the male-sterile C545 line without and after a 3 d heat treatment at 37 °C. (B) For Comparison 1 (C545 versus SORA 1), 8075 DEGs were identified and for Comparison 2 (C545 heat versus C545) 16 697 DEGs. The Venn diagram displays the 4029 DEGs that were common to both comparisons. DEGs were defined as those having a fold change of either ≥2 or ≤ − 2 with an adjusted *P*-value of ≤0.05. (C) Dot plot displaying the top 10 enriched GO terms from up- and down-regulated genes in the two comparisons. The size of the dots is proportional to the number of DEGs within that GO term. The colour indicates the adjusted *P*-value of the enriched GO term.

Three different RNA samples were isolated for this study and used in two different comparisons (Fig. 6A). For comparison, one RNA was isolated from 2 mm long unopened buds from both C545 and SORA 1 flowers (C545 versus SORA 1, Fig. 6A), representing a comparison between sterile and fertile buds. In the second comparison, a third RNA sample was isolated from C545 buds after a 3 d heat treatment at 37 °C, which represents a comparison of heat stress fertility-restored buds and male-sterile buds from the same genotype (C545 heat versus C545, Fig. 6A). Isolated RNA samples were sequenced using the Illumina platform and subjected to RNA-seq analysis. For the two comparisons, a total of 20 743 DEGs (log2-fold change ≥1 or ≤ −1 with an adjusted *P*-value of ≤0.05) were identified with 4029 common DEGs (Fig. 6B). The DEGs for Comparison one, C545 versus SORA 1, contained 3677 down-regulated and 4398 up-regulated genes (Supplementary Table S3). In Comparison two, C545 heat versus C545, we identified 8558 down-regulated and 8140 up-regulated genes (Supplementary Table S3), indicating that the heat treatment resulted in more changes to the transcriptome than between untreated male-sterile and fertile buds.

For an understanding of the biological significance of the gene expression changes, a GO analysis was performed on the down- and up-regulated DEGs from both comparisons (Fig. 6C; Supplementary Table S4). The GO analysis provides an insight into the important roles of enriched groups of genes in regulating male sterility and in restoring fertility in the C545 line. For the comparison of C545 versus SORA 1, the GO term enrichment analysis of the down-regulated DEGs displayed an enrichment of GO terms related to ‘fatty acid biosynthetic process’ including ‘l-phenyalanine catabolic process’, ‘*S*-adenosylmethioninamine biosynthetic process’, ‘phosphatidylcholine biosynthetic process’, ‘glucosinolate catabolic process’, ‘cinnamic acid biosynthetic process’, and ‘purine ribonucleoside salvage’ (Fig. 6C; Supplementary Table S4). This indicates that the chloroplast localization of BnChimera possibly leads to a disruption of fatty acid synthesis in chloroplasts, ultimately resulting in male sterility. Interestingly, other chloroplast GO terms including ‘photosystem I’ and ‘photosystem II’ also appear, which could indicate broader chloroplast metabolic impediments besides simply fatty acid synthesis (Fig. 6C; Supplementary Table S4). Also, polyamine biosynthesis appears to be down-regulated in C545 in comparison with SORA 1 (Fig. 6C; Supplementary Table S4). GO enrichment of the up-regulated DEGs in the C545 versus SORA 1 comparison demonstrated that buds in the C545 line are obviously stressed. Several stress-related GO terms are significantly enriched, including ‘response to chitin’, ‘systemic acquired resistance’, ‘response to oxidative stress’, ‘response to wounding’, ‘response to oomycetes’, ‘defence response to fungus’, ‘hydrogen peroxide catabolic process’, and ‘cellular oxidant detoxification’ (Fig. 6C; Supplementary Table S4). The finding of ‘anaerobic respiration’ in the up-regulated DEGs is possibly related to the fact that the microspores are constantly surrounded by a layer of thick callose, which may interfere with oxygen diffusion, possibly ending in the suffocation of the microspores leading to male sterility (Figs 1C, 6C; Supplementary Table S4).

GO enrichment analysis of the up- and down-regulated DEGs after heat treatment of the C545 line revealed some interesting results. For down-regulated DEGs, many GO terms were associated with the cell cycle, including ‘mitotic cell cycle phase transition’, ‘male meiosis II’, ‘mitotic cell cycle’, ‘cytokinesis by cell plate formation’, ‘resolution of meiotic recombination intermediates’, ‘preprophase band assembly’, ‘meiotic sister chromatid cohesion, centromeric’, and ‘homologous chromosome pairing at meiosis’ (Fig. 6C; Supplementary Table S4). This possibly indicates that cell cycle progression is inhibited or slowed down upon heat treatment, which could explain why pollen development is readily aborted during heat treatment in most plant species. A cell cycle arrest could also be linked to groups of GO terms enriched in ‘DNA replication initiation’, including ‘mitotic chromosome condensation’, ‘nucleosome positioning’, ‘DNA replication, synthesis of RNA primer’, and ‘mitotic DNA replication imitation’ (Fig. 6C; Supplementary Table S4). Interestingly, several GO terms associated with ‘regulation of cyclin-dependent protein serine/threonine kinase activity’ such as ‘regulation of cytokinesis’, ‘regulation of mitotic spindle organization’, and ‘mitotic spindle assembly checkpoint’ are also found within the down-regulated DEGs (Fig. 6C; Supplementary Table S4). The above, when also accompanied by a down-regulation of GO terms encompassing ‘sporopollenin biosynthetic process’, ‘pollen development’, ‘pollen exine formation’, ‘anther wall tapetum development’, and ‘anther development’ would all suggest that after heat treatment, pollen development should be inhibited or blocked, but is clearly not in C545 (Figs 1A, 6C; Supplementary Table S4). Strikingly, fatty acid biosynthesis appears to be down-regulated in the C545 line after heat treatment (Fig. 6C; Supplementary Table S4). Surprisingly, within the after heat treatment up-regulated DEGs, many of the same GO terms which were enriched in the C545 versus SORA 1 comparison are also found in the up-regulated DEGs of the C545 heat versus C545 comparison (Fig. 6C; Supplementary Table S4). Many of these enriched GO terms represent stress responses. These include heat stress-related GO terms ‘response to heat’ and ‘heat acclimation’. Other GO terms relate to ‘response to chitin’ or ‘defence response to oomycetes’ (Fig. 6C; Supplementary Table S4). This could indicate that, since the C545 line is already showing indications of stress, the buds are already primed to respond to the heat stress treatment.

### DEGs encoding chloroplast-targeted proteins

**Fig. 7.**
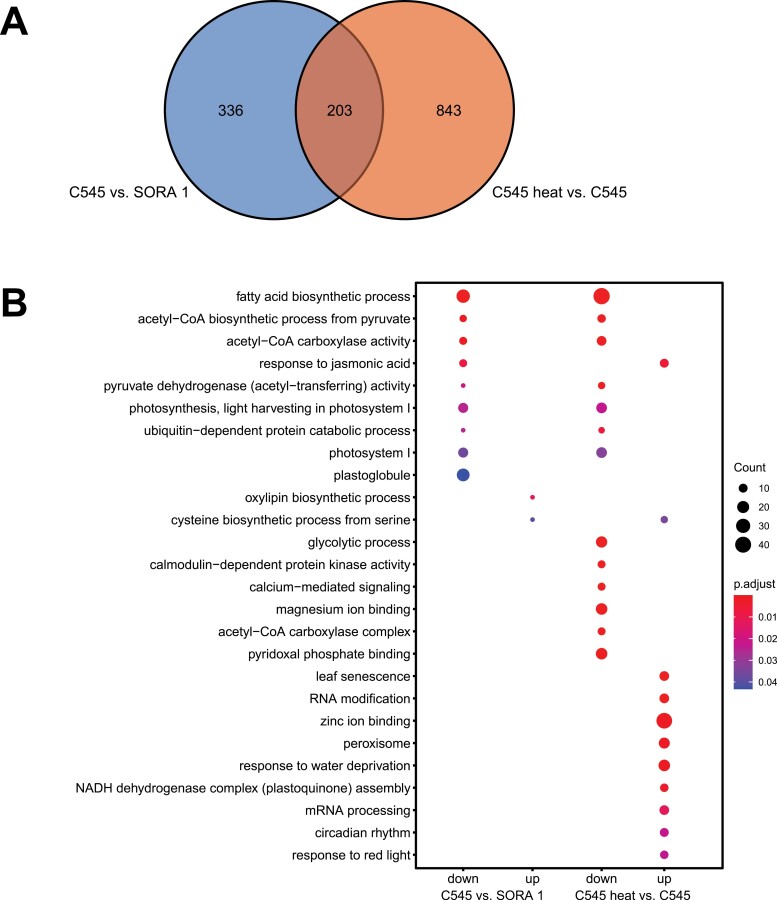
Analysis of DEGs coding for chloroplast proteins. (A) For the C545 versus SORA 1 comparison, 539 DEGs were identified, which encode chloroplast-targeted proteins. Between C545 heat-treated and C545, 1046 DEGs were identified that code for chloroplast-targeted proteins. The Venn diagram indicates that the two comparisons share 203 DEGs. (B) Dot plot displaying the top 10 enriched GO terms of DEGs coding for chloroplast-targeted proteins in the indicated comparisons.

Since it has now been established that both BnChimera and BnaC9-Tic40 interact and are localized to chloroplasts, a more detailed analysis of DEGs coding for chloroplast-targeted proteins was performed. To identify chloroplast-targeted proteins in *B. napus*, firstly a list of known chloroplast proteins from *A. thaliana* was prepared using data from the SUBA4 database (Hooper *et al.*, 2017). A *B. napus* protein was then considered as chloroplast localized if its top BLAST hit was from the Arabidopsis chloroplast list. This analysis identified a list of 4716 genes which potentially encode chloroplast-localized proteins in *B. napus.* Of these 4716 genes, 1382 genes were identified as DEGs in our dataset and 203 genes overlap between the two different comparisons (Fig. 7A; Supplementary Table S5). For the comparison of C545 versus SORA 1, 340 down- and 199 up-regulated DEGs encoding chloroplast-targeted proteins were identified (Supplementary Table S5). On the other hand, in the C545 heat versus C545 comparison, there are 471 down- and 575 up-regulated DEGS coding for chloroplast-targeted proteins (Supplementary Table S5). This again demonstrates a stronger influence of the heat treatment on gene expression than any difference between untreated male-sterile or fertile flowers.

GO enrichment analysis of DEGs coding for chloroplast-targeted proteins indicated that many of the same processes in chloroplasts are down-regulated in both comparisons (Fig. 7B; Supplementary Table S5). For example, in both the C545 versus SORA 1 and C545 heat versus C545 comparisons, GO terms such as ‘fatty acid biosynthetic process’, ‘acetyl-CoA biosynthetic process from pyruvate’, ‘photosynthesis, light harvesting in photosystem I’, and ‘photosystem I’ were over-represented (Fig. 7B; Supplementary Table S5). This indicates that in both the sterile untreated and heat-treated fertile flowers, although fatty acid synthesis is potentially the main site of inhibition, photosynthesis may also be perturbed, which is most probably a secondary effect (Fig. 7B, Supplementary Table S6). In reference to GO terms of the up-regulated DEGs, in the C545 versus SORA 1 comparison, only two enriched GO terms were identified while for the heat-treated comparison again several stress-related GO terms were found, including ‘response to water deprivation’, ‘leaf senescence’, ‘response to red light’, and ‘response to cold’ (Fig. 7B; Supplementary Table S6). When combining all these transcript data, what is lacking is a definitive chloroplast function in the down-regulated population of C545 versus SORA 1 and in the up-regulated population after the heat treatment, or vice versa. Not observing such a group makes it difficult to pinpoint the exact inhibition, which leads to either the male sterility in the C545 line or to the restoration of fertility after the heat treatment.

### Analysis of several affected functional groups

**Fig. 8.**
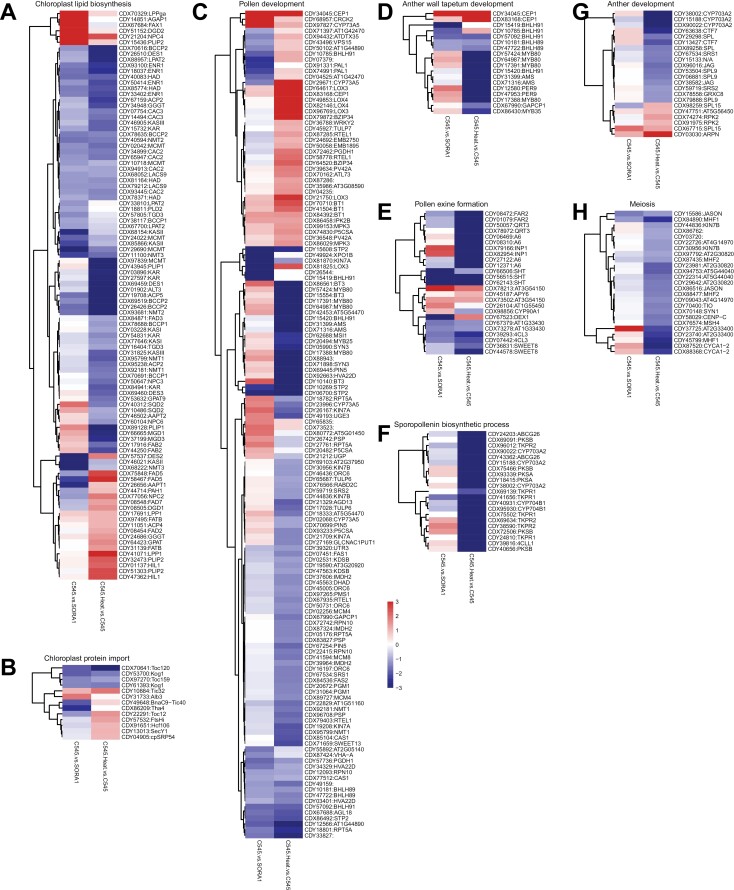
Hierarchical clustering of gene expression of selected processes. Shown are genes with a significant differential expression in one of the comparisons with (A) 99 DEGs out of a total of 230 genes involved in chloroplast lipid biosynthesis, (B) in chloroplast protein import with 13 DEGs out of 130 genes, (C) in pollen development with 144 DEGs out of 381 genes, (D) in anther wall tapetum development with 18 DEGs out of 25 genes, (E) in pollen exine formation with 25 DEGs out of 53 genes, (F) in the sporopollenin biosynthetic process with 21 DEGs out of 21 genes, (G) in anther development with 22 DEGs out of 49 genes, and (H) in meiosis with 25 DEGs out of 58 genes. See Supplementary Table S7 for full names of all genes.

Since no clear pathway reversion caused by the heat treatment was obvious, we looked closer at some more consistent changes in the transcriptome data. In both comparisons, fatty acid biosynthesis was down-regulated. Indeed, we identified all genes encoding proteins involved in chloroplast lipid biosynthesis to be differentially expressed in at least one of the comparisons. These chloroplast lipid biosynthesis genes were identified by taking the homologues of the previously published proteins (Holzl and Dormann, 2019). Out of 230 genes involved in chloroplast lipid biosynthesis, 99 genes were DEGs within the two comparisons (Fig. 8A; Supplementary Table S7). These 99 chloroplast lipid biosynthesis-associated DEGs were mostly down-regulated in both comparisons but more so after the heat treatment of C545 flowers (Fig. 8A). Therefore, lipid synthesis impairment could potentially be the reason for the male sterility as lipids are required for pollen cell wall formation, essential for pollen development and male fertility (Hater *et al.*, 2020). Because BnaC9-Tic40 is a chloroplast protein import component, we also analysed DEGs known to be involved in chloroplast protein import (Fig. 8B; Supplementary Table S7). However, only 13 DEGs were identified out of a possible 130 genes coding for chloroplast import components in the *Brassica* genome. This indicates that there are no major changes in the abundance of protein import components and that protein import into the chloroplast is not affected. As expected, in the C545 versus SORA 1 comparison, the transcript coding for BnaC9-Tic40 is also down-regulated, which is not surprising as it contains a premature stop codon and would produce a truncated non-functional protein (Fig. 8B; Supplementary Table S7).

One of the interesting findings from the GO term enrichment analysis was that many cell cycle-related GO terms were significantly enriched within the down-regulated DEGs in the C545 line after heat treatment (Fig. 6C; Supplementary Table S4). This may hint at a delay in pollen development within the flowers, slowed down or blocked after the heat treatment. Indeed, pollen development is considered as the most heat-sensitive stage in plant development (Lohani *et al.*, 2020). So, the DEGs from both comparisons were searched for genes related to the following selected GO terms: ‘pollen development’, ‘anther wall tapetum development’, ‘pollen exine formation’, ‘sporopollenin biosynthetic process’, ‘anther development’, and ‘meiosis’. Overwhelmingly, the majority of DEGs from these GO terms are down-regulated after the heat treatment of the C545 line (Fig. 8B–H; Supplementary Table S7). This is a little surprising considering that in some cases these genes are not down-regulated between male-sterile C545 and fertile SORA 1 plants. Within these down-regulated cell cycle genes, developmental DEGs are homologues of some well-known genes related to anther and pollen development including: *ABORTED MICROSPORES* (*AMS*) (Sorensen *et al.*, 2003), *MYB80 transcription factor* (*MYB80*) (Phan *et al.*, 2011), *MYB35* (Zhu *et al.*, 2008), *cysteine protease CEP1* (Zhang *et al.*, 2014), *basic helix–loop–helix proteins 89 and 91* (*bHLH89* and *bHLH91*) (Zhu *et al.*, 2015), *DEFECTIVE IN EXINE FORMATION 1* (*DEX1*) (Paxson-Sowders *et al.*, 2001), and *cytochrome P450* (*CYP703A2*) (Morant *et al.*, 2007) (Fig. 8C–F; Supplementary Table S7). This illustrates that in the C545 line after heat treatment, counterintuitively anther and pollen developmental genes are down-regulated, which, when combined with a potential lipid synthesis bottleneck, may lead to fertility restoration.

## Discussion

The MSL system from Europe and the 9012AB/7365ABC system from China are two of the world’s main pollination control systems for hybrid rapeseed production (Chen *et al.*, 1993; Frauen *et al*., 2003, 2006). Even though rapeseed is the second most economically important edible oil seed crop, the identity of the genes for both male sterility (*MS*) and restorer of fertility (*Rf*) in either system remained unknown until recently. In fact, both systems seemed to have the same temporary maintainer system and showed very similar morphological observations during pollen abortion (Luo *et al.*, 2018). In this study, we present data that show that both the MSL system and 9012AB share the same *MS* gene called *BnChimera* (also known as *Bnrf*^*b*^), which encodes a chloroplast-targeted chimeric protein (Dun *et al*., 2011, 2014; Li *et al.*, 2012; Deng *et al.*, 2016; Xia *et al.*, 2016; Z. Zhang *et al.*, 2020). Using Arabidopsis, it could be demonstrated that *BnChimera* requires all its domains to cause male sterility. The restorer of fertility gene in both systems was shown to be *BnaC9-Tic40*. However, in contrast to previous work (Xia *et al.*, 2016), we found a direct and specific interaction between the BnChimera and BnaC9-Tic40 proteins. Interestingly, both systems display a heat stress-reversible male fertility, which we attribute to a slowdown of pollen development. Our results also led us to offer an alternative hypothesis regarding how *BnChimera* causes male sterility, about how *BnaC9-Tic40* rescues male fertility, and about how heat stress may also revert the male-sterile phenotype.

### How does the male sterility system work in the MSL and 9012AB lines?

In previous work on the 9012AB male sterility system, an extremely complicated explanation about how the system functions was proposed (Dun *et al*., 2011, 2014; Li *et al.*, 2012; Deng *et al.*, 2016; Xia *et al.*, 2016; Z. Zhang *et al.*, 2020). The explanation was that the chloroplast-targeted BnChimera interacts with the nuclear-localized E3 ligase BTS and thereby redirects BTS to the chloroplast outer envelope membrane. There, it is thought to affect chloroplast protein translocation by disrupting the normal ubiquitin–proteasome system causing toxic effects within the chloroplast, ultimately resulting in male sterility (Z. Zhang *et al.*, 2020). These defects can be overcome via either heat treatment, which suppresses the interaction between BnChimera and BTS as demonstrated in yeast two-hybrid experiments (Z. Zhang *et al.*, 2020), or via an interaction between BnaC9-Tic40 and Toc33. This interaction then sends a signal to the nucleus to inhibit *BnChimera* expression or to remove the BnChimera protein through an unknown mechanism, thus eliminating its toxic effects (Z. Zhang *et al.*, 2020). Here, we would like to offer a more straightforward hypothesis about how these and the MSL system function based on our own and previously published results.

**Fig. 9.**
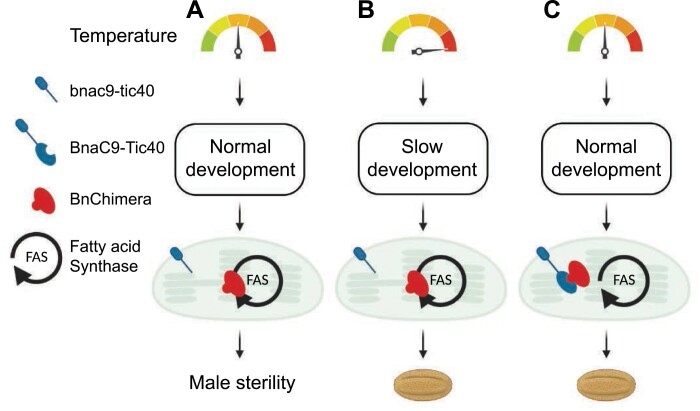
Predicted model for how heat treatment restores male fertility in C545. (A) The C545 line under normal conditions is male sterile because *bnac9-tic40* is truncated at the C-terminus and therefore unable to interact with BnChimera. This allows BnChimera to disrupt, block, or inhibit fatty acid synthesis within chloroplasts. Male sterility results from chloroplasts’ inability to produce enough fatty acids in time for the rapid pollen development. (B) At higher temperatures, pollen development is severely slowed down, which in turn provides the chloroplasts with time to produce enough lipids, restoring male fertility. (C) This is equivalent to the restorer line situation, in which BnaC9-Tic40 contains its complete C-terminus and can interact with BnChimera. In this situation, the flowers are male fertile as the interaction with BnaC9-Tic40 blocks the BnChimera-mediated inhibition of fatty acid synthesis. Created with BioRender.com.

Firstly, there is no evidence in any previously published work or in this study that the BnChimera is located at the outer envelope of chloroplasts. In fact, the GFP results presented here and in previous work would support a stromal location for BnChimera. In most cases, outer envelope proteins generally display a halo-like pattern around the chloroplast (Breuers *et al.*, 2012; Teresinski *et al.*, 2019). However, in our analysis and in those of others (Xia *et al.*, 2016), the BnChimera GFP pattern is rather diffuse and distributed within chloroplasts more like a stromal protein. In fact, BnChimera does not contain any predicted transmembrane domains, which makes it unlikely that BnChimera could interact *in vivo* with anything on the outer envelope of chloroplasts or with a nuclear-localized protein. More plausible is that BnChimera is targeted to the stroma where it interacts with currently unidentified protein(s) that results in the disruption of, for example, fatty acid synthesis which would lead to male sterility (Fig. 9A). Disruption of processes affecting plastid fatty acid synthesis and export have been previously demonstrated to cause male sterility in plants (Li *et al.*, 2015; T. Zhu *et al.*, 2020; Zhang *et al.*, 2021). A possible involvement of BnChimera in lipid biosynthesis was previously proposed after conducting cutin and wax measurements (Xia *et al.*, 2016). A disruption of fatty acid biosynthesis caused by BnChimera is also supported by our RNA-seq experiments, since down-regulated DEGs were enriched in processes of fatty acid and lipid biosynthesis. It is also possible that chloroplast membrane lipid trafficking is affected, for example *FAX1*, which is responsible for the export of fatty acids from plastids, is heavily up-regulated in C545 (Li *et al.*, 2015). Ultimately, this disruption of fatty acid synthesis or export from plastids leads to male sterility due to the inability to synthesize any lipids required for the pollen cell wall (Fig. 8A).

Secondly, we could demonstrate that the restorer gene product BnaC9-Tic40 directly interacts with BnChimera. Since this involves the C-terminus of BnaC9-Tic40, which is known to face the stroma, this also strongly indicates a stromal location of BnChimera. A direct interaction between the *MS* and *Rf* gene products allows a more clear-cut explanation for the restoration of male fertility. Since in the male-sterile lines *bnaC9-tic40* contains a premature stop codon, the protein is missing its C-terminus, which is responsible for the interaction with BnChimera (Dun *et al.*, 2011). Our hypothesis here is that, when full-length BnaC9-Tic40 is present, it interacts with BnChimera within the stroma of chloroplasts and prevents BnChimera from interfering with fatty acid synthesis (Fig. 9C). This may probably be just by way of competition. Since out of the four Tic40-like proteins encoded within the *B. napus* genome only BnaC9-Tic40 can interact with BnChimera, it is the only one which can confer the restorer function. Interestingly, the amino acid phenylalanine at position 321, which was demonstrated to be essential for the restorer function of BnaC9-Tic40, is also essential for the interaction with BnChimera (Fig. 4B) (Xia *et al.*, 2016; Z. Zhang *et al.*, 2020). It must also be pointed out that any interaction between Toc33 and Tic40 in the outer envelope membrane is not under scrutiny here. There is no evidence for any Tic40 being located within the outer envelope, and it is considered as an inner envelope membrane marker protein with its C-terminus facing the stroma (Stahl *et al.*, 1999; Bedard *et al.*, 2007; Chou *et al.*, 2003). Therefore, it is extremely unlikely that the C-terminus of BnaC9-Tic40 interacts *in vivo* with Toc33.

### How does heat stress revert male sterility?

Since BnChimera is unlikely to be localized to the outer envelope of chloroplasts, heat stress inhibition of the interaction with BTS is also unlikely. In our observations, transcript abundance of *BnChimera* after heat treatment does not significantly change (Supplementary Fig. S6). It is assumed that even after heat treatment, the BnChimera protein is still found in chloroplasts; however, it cannot be completely ruled out that the BnChimera is unstable after heat treatment or that its interaction with other proteins is disrupted. Therefore, we offer an alternative hypothesis about how heat treatment reverses male sterility.

The developmental process of pollen, including microgametogenesis, depends on a strict and timely coordination of meiosis, mitosis, cell growth, and expansion (Sanders *et al.*, 1999). One of the reasons why BnChimera causes male sterility is that during the relatively fast development of pollen, chloroplasts cannot synthesize sufficient material for pollen wall formation, which ultimately leads to defective microspores and male sterility. These effects are then reversed via heat treatment. From the RNA-seq analysis of the C545 male-sterile line, the largest group of down-regulated DEGs after heat treatment is involved in cell cycle progression including meiosis and mitosis, as well as in pollen development. It has been demonstrated before that heat stress affects pollen meiosis (Sakata *et al.*, 2000). Therefore, we hypothesize that during heat stress pollen development is slowed down, which allows chloroplasts more time to synthesize sufficient material for pollen cell wall development. This combination of slower development and slower synthesis of cell wall material results in fertile pollen (Fig. 9B). A similar phenomenon was recently described in Arabidopsis TGMS lines, in which lower temperatures restored fertility by slowing pollen development (C. Zhang *et al.*, 2020; J. Zhu *et al.*, 2020). Interestingly, in one of these studies, the authors concluded that, at least in Arabidopsis, heat treatment did not restore fertility but reduced it (C. Zhang *et al.*, 2020). These experiments, however, were performed on Arabidopsis T-DNA insertional mutants, whereas in rapeseed the mutation is not a knockout.

The development of the male reproduction system in plants has been demonstrated many times to be extremely sensitive to adverse environmental conditions (De Storme and Geelen, 2014). This includes both heat and cold stress whereby heat stress is typically observed to show the premature disappearance of the tapetal cell layer in conjunction with microspore development issues (Ku *et al.*, 2003; Abiko *et al.*, 2005; Oshino *et al.*, 2007). Cold stress, on the other hand, displays an almost opposite effect in that the tapetum persists right up until the mature pollen stage (Mamun *et al.*, 2006; Oda *et al.*, 2010). In both cases, the environmental temperature results in male sterility; however, both heat and cold stress have also been demonstrated to cause male fertility restoration (Fernandez-Gomez *et al.*, 2020; C. Zhang *et al.*, 2020; J. Zhu *et al.*, 2020). Heat stress fertility restoration was recently shown to be important at an early stage of pollen development, more specifically prior to pollen mitosis I (Fernandez-Gomez *et al.*, 2020). This may be relevant to the C545 line analysed here as the DEGs were enriched for cell division machinery in flowers heat treated early on in flower development. This could be the developmental slowdown we have hypothesized. Cold stress, on the other hand, leads to an overall slowdown of development not just on any specific pathways (C. Zhang *et al.*, 2020; J. Zhu *et al.*, 2020). It also leads to tapetum layer retention, which is already evident in the C545 sterile flowers, so cold treatment may only compound the tapetum developmental problems at normal growth temperatures. Heat stress was also demonstrated to lead to chloroplast overdevelopment (Oshino *et al.*, 2007). Therefore, it could also be hypothesized that in the C545 line the BnChimera could block chloroplast development at normal growth temperatures, but chloroplast development can then be induced during the heat stress; something to consider since the BnChimera is localized to chloroplasts. However, since the observed changes in gene expression after heat treatment support a reduction in transcripts related to cell cycle and cellular division, it is possible that the short (3 d) heat treatment leads to a temporary halt or slowing down of pollen development most probably related to cellular division, which allows the perturbed chloroplasts more time to synthesize sufficient material for pollen cell wall development.

### Potential for using BnChimera to create thermotolerant pollen

Due to the adverse effects of climate change, pollen thermotolerance is becoming an increasingly important economic trait for breeding. Therefore, the ability of the C545 line to produce seeds at high temperatures potentially could be used to help create thermotolerant pollen. This means that understanding how the MSL system can produce viable pollen after heat treatment becomes more relevant. The ability of the C545 line to survive heat could be related to the genes we identified as up-regulated DEGs in the C545 line in comparison with SORA 1. The vast majority of these DEGs were related to various stress responses. Due to this large number of stress-related up-regulated DEGs, the C545 line is already primed for responding to stress. Similar observations have been made before whereby the adverse effects of heat stress can be circumvented to a certain extent when plants experience a ‘pre-conditioning treatment’ (Chaturvedi *et al.*, 2021). This is normally achieved by exposing plants to a mild stress treatment, which is followed by a brief recovery period whereby an acquired thermotolerance is induced, allowing the plants to survive a normally lethal heat stress (Chaturvedi *et al.*, 2021). The capacity for plants to acquire thermotolerance has been attributed to the ability of cells to produce and store certain proteins, which enhance their resistance to higher temperatures (Larkindale and Vierling, 2008). This was recently demonstrated for tomato plants, in which heat treatment of 50 °C results in reduced pollen germination rates. However, if plants are pre-treated for a brief period at 32 °C followed by a short recovery phase at 25 °C, this enhanced the tolerance to 50 ° C (Firon *et al.*, 2012; Jegadeesan *et al.*, 2018). Of course, thermotolerance is not limited to reproductive tissues, as a gradual increase in temperature versus a sudden 40 °C heat treatment of seeds also showed that the slower acclimation increased thermotolerance (Stone and Nicolas, 1995). In the MSL system, the expression of the full-length BnChimera not only produces sterile pollen, but also induces enough changes within the transcriptome, which equate to a stress pre-treatment. Thus, if the expression of the BnChimera could be used to induce this pre-treatment effect without causing male sterility, this would be invaluable. Potentially using the fragments of BnChimera, as we have here, could produce a similar transcriptome stress response but no male sterility, which warrants further experimental work. Alternatively, BnChimera could be transformed into other crop plants for inducing stress responses and then the BnaC9-Tic40 could be transiently expressed via the newly developed spray-on viral transfection technology to produce viable pollen (Massel *et al.*, 2021; Torti *et al.*, 2021). This would allow a tuneable expression and control of pollen development.

In conclusion, this study has identified the *MS* gene of the MSL system and demonstrated that the regulation of the MSL system occurs within chloroplasts. Furthermore, using RNA-seq provided new insights into the thermosensitive male sterility system, displaying a high temperature fertility restoration. Overall, the results may prove useful for future plant breeding strategies as the MSL sterile lines display signs of a conditional pre-treatment to stress, potentially allowing the plants to survive heat. When combined with the need to produce crop plants, which are resistant to a future warming climate, these findings could be used to design plants which display thermotolerance during pollen development.

## Supplementary data

The following supplementary data are available at *JXB* online. 

Fig. S1. Light microscopy of developing anthers from SORA 1 and C545 before and after heat treatment.

Fig. S2. Identification of *BnaC9-Tic40* as the restorer gene of the MSL system.

Fig. S3. Determination of the existence of the *MS* gene in different lines of the MSL system.

Fig. S4. BnChimera expression analysis from yeast.

Fig. S5. 2D-NMR evaluation of the Tic40 variants A10 and C09.

Fig. S6. qRT–PCR of the *BnChimera* transcript before and after heat treatment.

Table S1. Primers used in the study.

Table S2. Assembled contigs of clone BAC3 and physical positions of the *Rf*-linked markers.

Table S3. List of differentially expressed genes.

Table S4. GO enrichment data.

Table S5. List of differentially expressed genes encoding chloroplast-targeted proteins.

Table S6. GO enrichment data for DEGs encoding chloroplast-targeted proteins.

Table S7. List of DEGs used in the heatmaps in Fig. 7.

erac082_suppl_supplementary_table_S1

erac082_suppl_supplementary_table_S2

erac082_suppl_supplementary_table_S3

erac082_suppl_supplementary_table_S4

erac082_suppl_supplementary_table_S5

erac082_suppl_supplementary_table_S6

erac082_suppl_supplementary_table_S7

erac082_suppl_supplementary_figures_S1-S6

## Data Availability

The RNA-seq data were deposited in the NCBI Sequence Read Archive (SRA) (https://www.ncbi.nlm.nih.gov/sra) with the Project ID PRJNA755517.

## Abbreviations

CD: circular dichroism
DEG: differentially expressed gene
GFP: green fluorescent protein
GMS: genetic male sterility
MSL: male sterility Lembke system
NPZ: Norddeutsche Pfanzenzucht Hans-Georg Lembke
*Rf*: fertility restorer gene
ROS: reactive oxygen species
TFE: trifluoroethanol
TGMS: temperature-sensitive genic male sterility
TIC: translocon of the inner chloroplast membrane
TOC: translocon of the outer chloroplast membrane
