# Supplementary material for: Two wrongs make a right: heat stress reversion of a male-sterile Brassica napus line
Source: J Exp Bot. 2022 Feb 28;73(11):3531–51. doi: 10.1093/jxb/erac082 (PMC9162185; doi:10.1093/jxb/erac082)
Supplement: erac082_suppl_supplementary_figures_S1-S6 [file erac082_suppl_supplementary_figures_s1-s6.pdf]

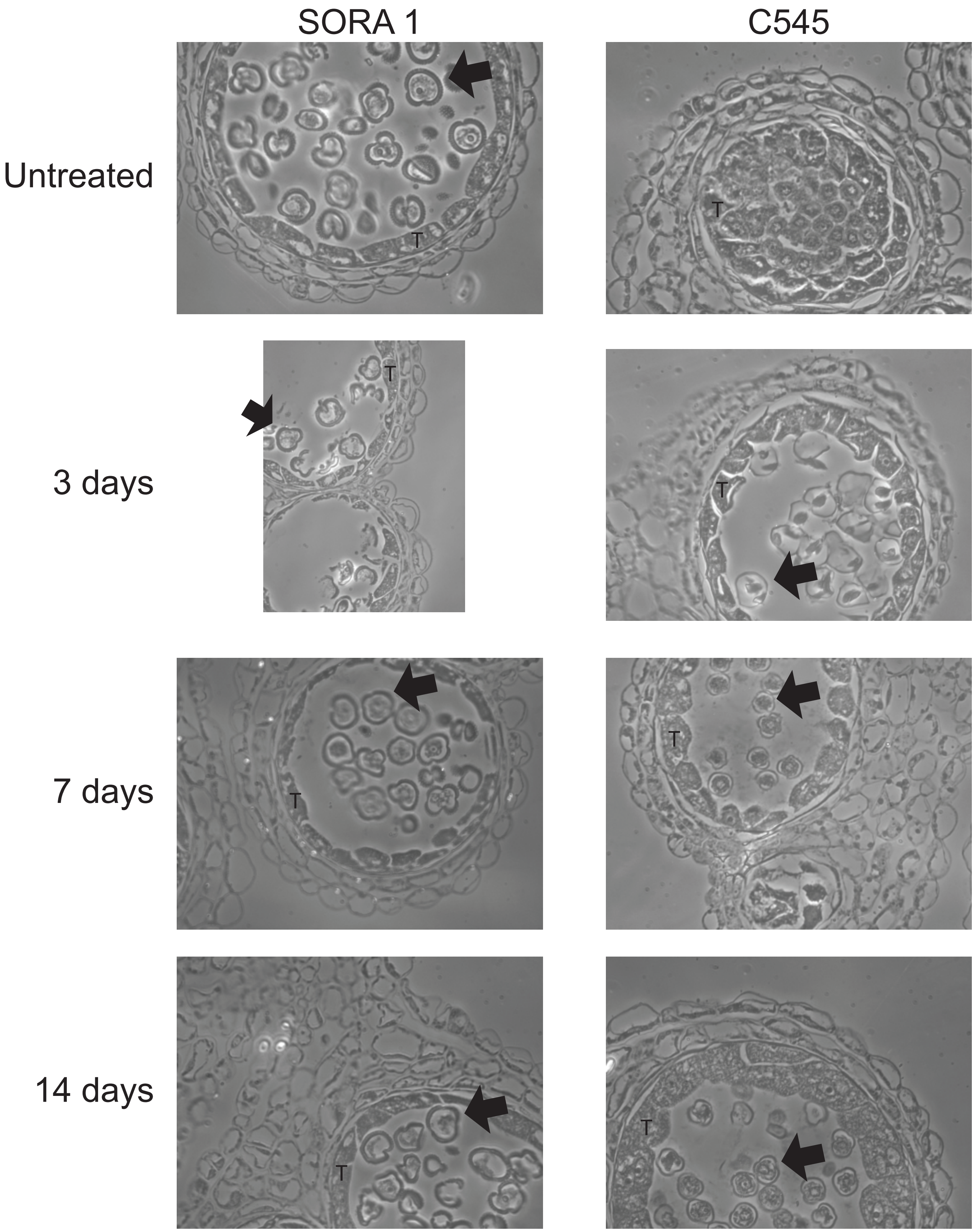

Supplemental Figure 1. Light microscopy of developing anthers from SORA 1 and C545 before and after heat treatment. Light microscopy images of semi-thin sections (1-2 µm) from representative anthers for 2 mm long buds from SORA 1 and C545 lines. Buds are at stages 9-10 of development. The number of days indicates the length of heat treatment at 37 °C. Arrows indicate the same released mature microspores from Figure 1. T = Tapetum

Supplemental Figure 1.

A

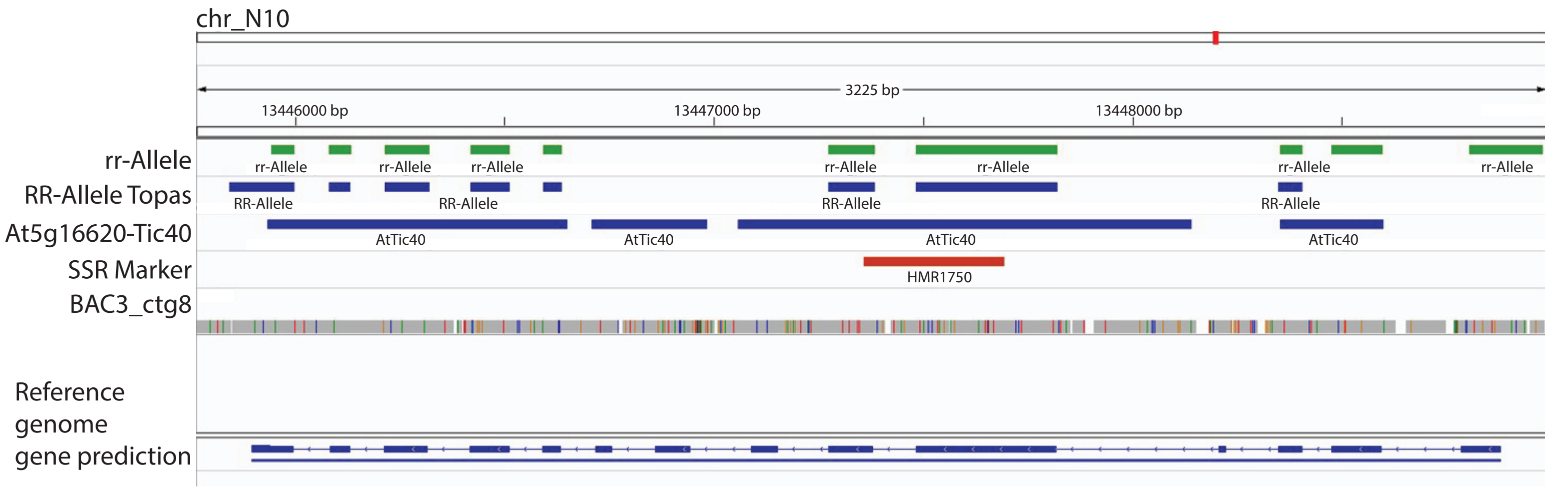

B

```
A-Line     c a g c c t c a a t c c c a g t c t t c a g g t g c t a c t g t t g a t g t g a c a g c t a c a a a   50
C545       c a g c c t c a a t c c c a g t c t t c a g g g g c t a c t g t t g a t g t g a c a g c t a c a a a   50
SORA1      c a g c c t c a a t c c c a g t c t t c a g g t g c t a c t g t t g a t c t g a c a g c t a c a a a   50
Published  c a g c c t c a a t c c c a g t c t t c a g g t g c t a c t g t t g a t g t g a c a g c t a c a a a   50

A-Line     a g t a g a t a g g c c t c c t g t g t c t a a g c c a c a a c c t a c a c c t a t - - c t c c t a   98
C545       a g t a g a t a g g c c t c c t g t g t c t a a g c c a c a a c c t a c a c c t a t - - c t c c t a   98
SORA1      a g t a g a t a g g c c t c c t g t g t c t a a g c c a c a a c c t a c a c c t a t a c c t c c t a  100
Published  a g t a g a t a g g c c t c c t g t g t c t a a g c c a c a a c c t a c a c c t a t a c c t c c t a  100

A-Line     c a a a g a g c a t a g a a g t g t a t a a a c c a a g t g t t g t c g t a g a g g a a g a c a a a  148
C545       c a a a g a g c a t a g a a g t g t a t a a a c c a a g t g t t g t c g t a g a g g a a g a c a a a  148
SORA1      c a a a g a g c a t a g a a g t g t a t a a a c c a a g t g t t g t c g t a g a g g a a a a c a a a  150
Published  c a a a g a g c a t a g a a g t g t a t a a a c c a a g t g t t g t c g t a g a g g a a g a c a a a  150

A-Line     g c g a t g a a a g a a g a a a a g a a c t a c g g t a g a t t c t t a c c t c c t c t t g t g a c  198
C545       g c g a t g a a a g a a g a a a a g a a c t a c g g t a g a t t c t t a c c t c c t c t t g t g a c  198
SORA1      g c g a t g a a a g a a g a a a a g a a c t a c g g t a g a t t c t t a c c t c c t c t t g t g a c  200
Published  g c g a t g a a a g a a g a a a a g a a c t a c g g t a g a t t c t t a c c t c c t c t t g t g a c  200

A-Line     c g c  201
C545       c g c  201
SORA1      c g c  203
Published  c g c  203
```

Supplemental Figure 2. Identification of BnaC9-Tic40 as the restorer gene of the MSL system. A) Physical alignment of non-restorer (rr) and restorer (RR) alleles in relation to the Arabidopsis Tic40 gene (At5g16620) and the in-gene SSR-marker HMR1750, which was used for the detection of the relevant region on BAClone3, contig 8. HMR1750 enables the discrimination between rr and RR alleles. The predicted gene structure of the gene of the respective region within the used reference genome is shown at the bottom. **B)** Confirmation of the 2 bp deletion in BnaC9-Tic40 the A- and C545-Lines of the MSL system via sequencing of the PCR product obtained with the RCP170 primer pair. Red box indicated the 2 bp deletion.

Supplemental Figure 2.

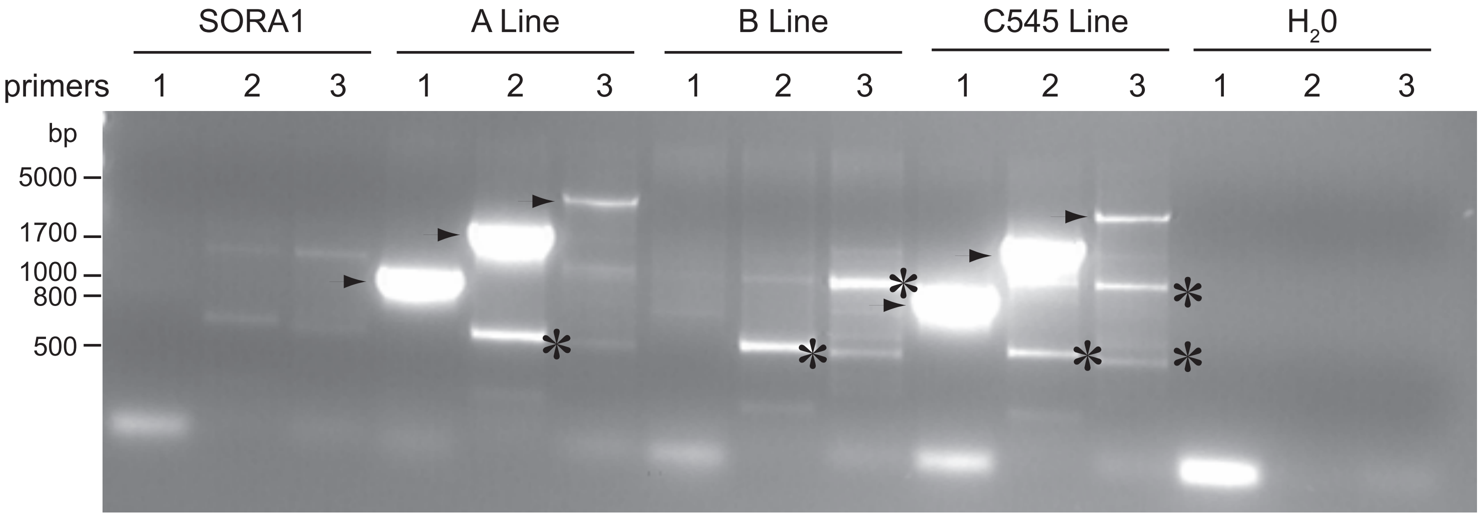

Supplemental Figure 3. Determination of the existence of the MS gene in different lines of the MSL system. DNA fragments found after the amplification from genomic DNA isolated from the indicated MSL system lines using the primer pairs HY1, HY2, and HY3. Arrow heads indicate the expected correct PCR products whereas the asterisks indicate non-specific PCR products.

Supplemental Figure 3.

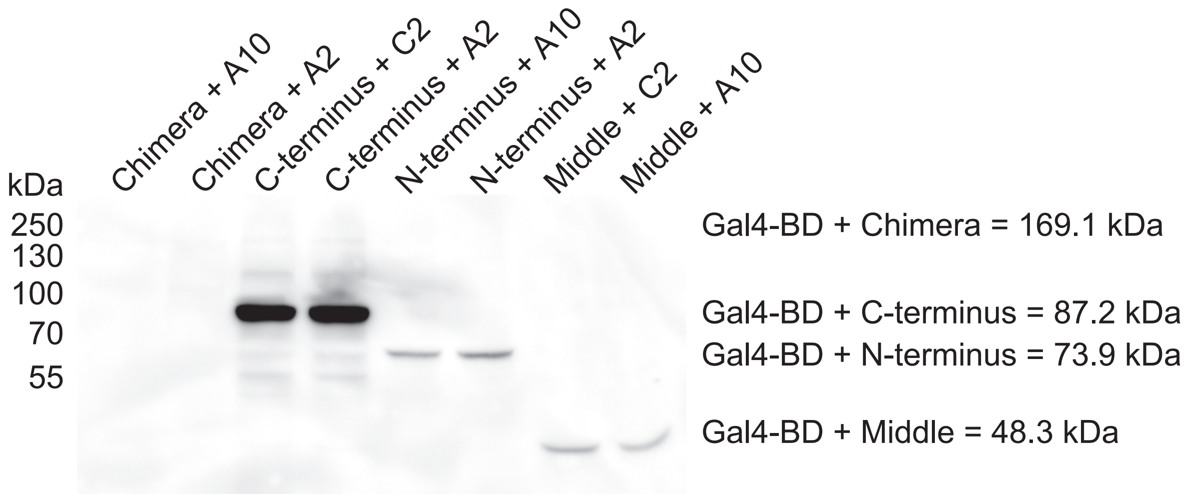

Supplemental Figure 4. BnChimera expression analysis from yeast. Whole protein extracts from the indicated yeast mating combinations were separated via SDS-gel electrophoresis, transferred to a PVDF membrane, and blotted using an antibody against the c-Myc tag. On the right, information of the predicted protein molecular weights of each of BnChimera constructs are provided in kDa. On the left is the molecular weight marker in kDa.

Supplemental Figure 4.

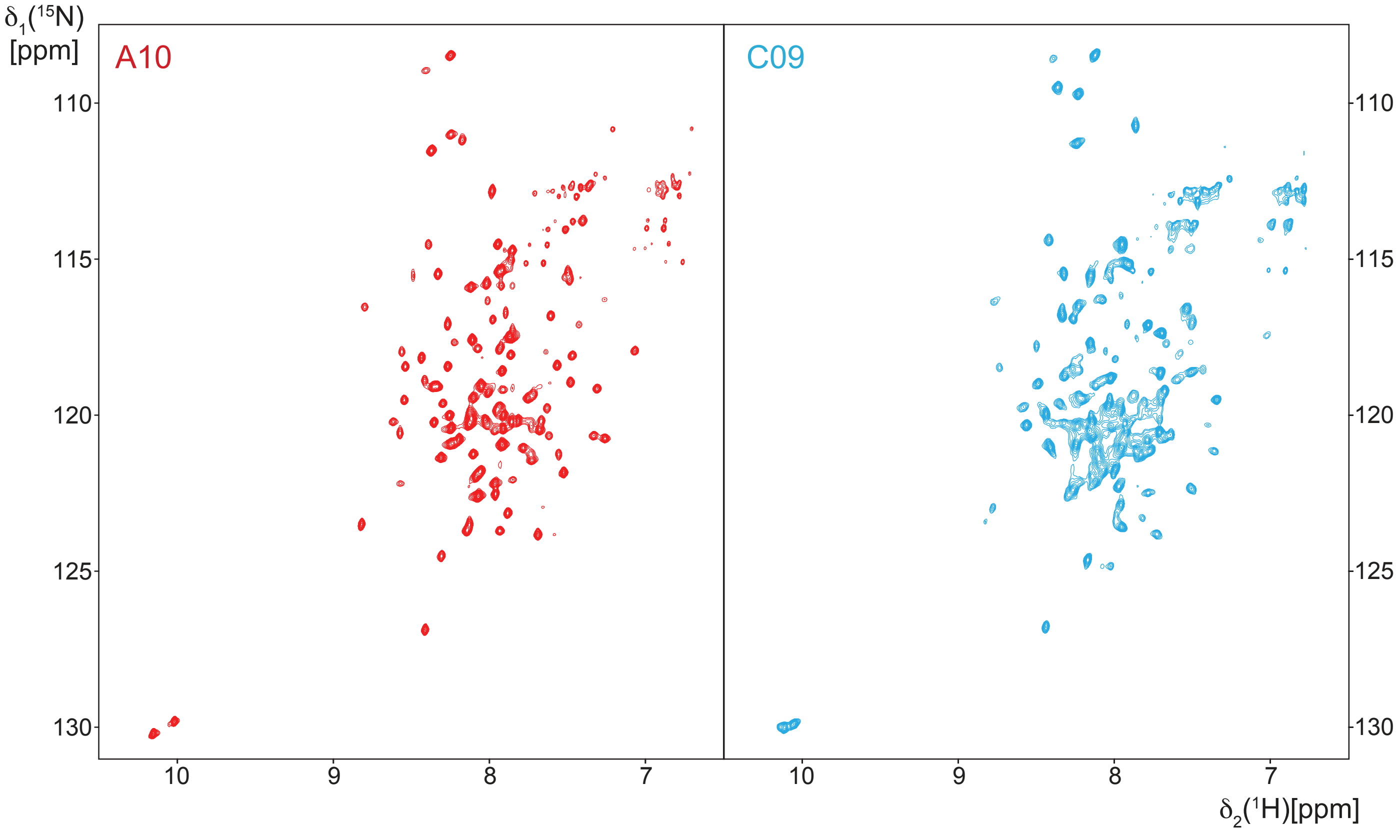

Supplemental Figure 5. 2D-NMR evaluation of the Tic40 variants A10 and C09. 2D-[^{15}N,^{1}H]-TROSY NMR spectra of 200 µM ^{15}N-labeled Tic40 A10 (left) and C09 (right) at 313K in 20 mM NaPi pH 6.0, 50 mM NaCl, 0.5 mM EDTA, 1 mM TCEP, 7 % D_2O. The spectral quality of the C09 variant is markedly decreased as compared to the A10 variant.

Supplemental Figure 5.

A

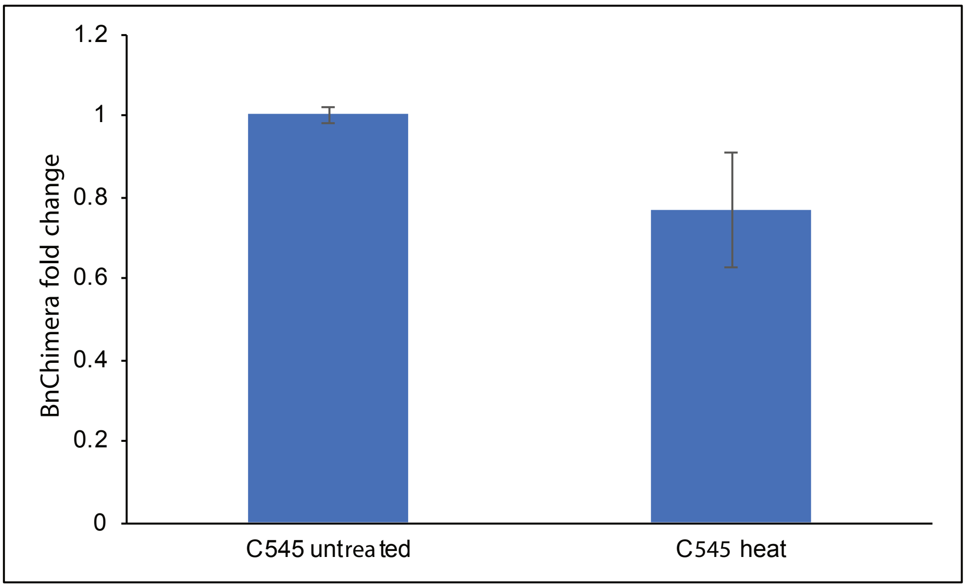

B

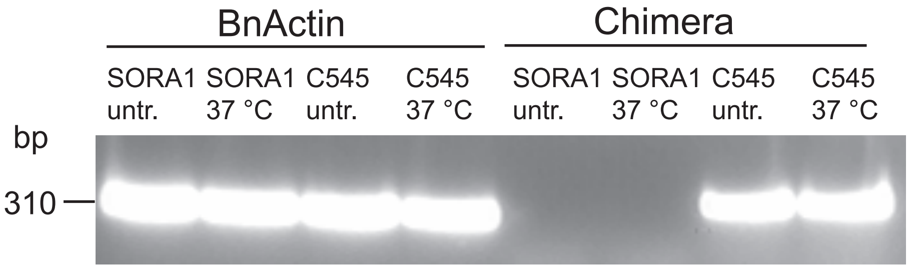

Supplemental Figure 6. qRT-PCR of the BnChimera transcript before and after heat treatment. A) qRT-PCR results of the expression levels of the MS gene BnChimera in the untreated C545 line and treated at 37 °C. BnActin and BnTic40-A2 were used as references. The calculation of the fold changes was performed using the $2^{\Delta\Delta\text{-ct}}$ method. **B)** Separation of the PCR products produced by the qRT-PCR via agarose gel electroporation. There is no expression of the BnChimera in the SORA 1 line, supporting its absence from the genome.

Supplemental Figure 6.
